# Accurate lineshape spectroscopy and the Boltzmann constant

**DOI:** 10.1038/ncomms9345

**Published:** 2015-10-14

**Authors:** G.-W. Truong, J. D. Anstie, E. F. May, T. M. Stace, A. N. Luiten

**Affiliations:** 1 Institute for Photonics and Advanced Sensing (IPAS) and School of Chemistry and Physics, The University of Adelaide, Adelaide, South Australia 5005, Australia; 2 School of Physics, The University of Western Australia, Perth, Western Australia 6009, Australia; 3 Centre for Energy, School of Mechanical and Chemical Engineering, The University of Western Australia, Crawley, Western Australia 6009, Australia; 4 ARC Centre for Engineered Quantum Systems, University of Queensland, Brisbane 4072, Australia

## Abstract

Spectroscopy has an illustrious history delivering serendipitous discoveries and providing a stringent testbed for new physical predictions, including applications from trace materials detection, to understanding the atmospheres of stars and planets, and even constraining cosmological models. Reaching fundamental-noise limits permits optimal extraction of spectroscopic information from an absorption measurement. Here, we demonstrate a quantum-limited spectrometer that delivers high-precision measurements of the absorption lineshape. These measurements yield a very accurate measurement of the excited-state (6P_1/2_) hyperfine splitting in Cs, and reveals a breakdown in the well-known Voigt spectral profile. We develop a theoretical model that accounts for this breakdown, explaining the observations to within the shot-noise limit. Our model enables us to infer the thermal velocity dispersion of the Cs vapour with an uncertainty of 35 p.p.m. within an hour. This allows us to determine a value for Boltzmann's constant with a precision of 6 p.p.m., and an uncertainty of 71 p.p.m.

## Abstract (web summary)

Reaching fundamental noise limits permits optimal extraction of spectroscopic information from an absorption measurement. Here, the authors demonstrate a quantum-limited spectrometer with which they can obtain an extremely accurate measurement of the excited-state hyperfine splitting in Cs.

## Graphical abstract

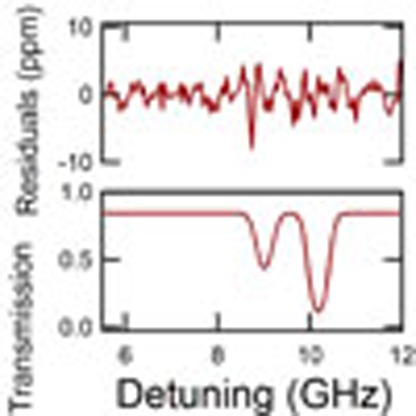

Spectroscopy is a vital tool for both fundamental and applied studies. Improving both the precision and accuracy of spectroscopic measurements is critical to efforts targeted at gaining maximum physical information about the quantum absorbers—even as they are perturbed by the probe. The immediate motivation for our work has arisen out of a call to the scientific community to develop new techniques to re-measure Boltzmann's constant, *k*_B_, in preparation for a redefinition of the kelvin^1^; however, advances in absorption lineshape measurement and theory will find applications in accurate gas detection and monitoring^2^, studies of planetary atmospheres^3^, thermometry in tokamaks^4^ and understanding distant astrophysical processes^5^,^6^. The accurate measurement of natural linewidths and transition frequencies, which can be directly related to the atomic lifetime, level structure and transition probabilities, is important for testing atomic physics^7^,^8^,^9^.

At thermal equilibrium, the velocity distribution of atomic absorbers in a vapour cell is related to the temperature through the Boltzmann distribution. This simple and fundamental relationship forms an excellent foundation for a type of primary thermometry known as Doppler broadening thermometry (DBT)^10^,^11^. DBT differs from the current leading technique for primary thermometry, which measures the temperature-dependent speed of sound in a noble gas contained in a well-characterized acoustic resonator. Using that approach, a breakthrough determination of *k*_B_ was made in 1988 with a total uncertainty of 1.7 p.p.m. (ref. 12). Refinements to this technique over the last 20 years have reduced its uncertainty to the 1 p.p.m. level^13^,^14^, improving the uncertainty in the CODATA-recommended value for *k*_B_ from 1.8 p.p.m. (CODATA-02) to 0.91 p.p.m. (CODATA-10). Despite these superb measurements, it is important that different techniques are employed to measure *k*_B_ to reduce the possibility of underestimating systematic uncertainties that may be inherent to any single technique^15^.

The DBT approach is one of the most theoretically transparent of the new approaches to primary thermometry. The spectrum of a gas sample is measured with high precision, and a theoretical model is fitted to the measured data. If the model includes all of the relevant physics then one can accurately extract the contribution to spectral broadening arising solely from the thermal distribution of atomic/molecular speeds. Ammonia probed by a frequency-stabilized CO_2_ laser at 1.34 μm was the first thermometric substance employed in a DBT experiment^16^. Subsequently, an extended-cavity diode laser at 2 μm was used to probe a ro-vibrational transition of CO_2_ (ref. 17). In these first experiments, the lineshape was assumed to be a Gaussian or a Voigt profile (a Gaussian convolved with a Lorentzian). Since 2007, with the ambitious goal of approaching 1 p.p.m. accuracy, substantial experimental and theoretical improvements have been made to DBT using ammonia^18^,^19^, oxygen^20^, ethyne^21^ and water^22^. However, one key challenge peculiar to molecular absorbers is the need to account for complex collisional effects on the lineshape^19^. We avoid this by using a dilute atomic vapour^23^,^24^ with a strong dipole transition for which a tractable, microscopic theory has been developed^25^,^26^. The benefits of a range of different atomic species have been considered in ref. 27.

Here we present measurements of a transmission lineshape of cesium (Cs) vapour with a quantum-limited transmission uncertainty of 2 p.p.m. in a 1 s measurement, which is to our knowledge, a factor of 16 times superior to anything previously demonstrated^28^. This extreme precision, allows us to directly detect subtle lineshape perturbations that have not been previously observed. This observation prompted the development of a theoretical model, which now allows us to discriminate between the internal atomic state dynamics and their external motional degrees. Using the model we are able to estimate the velocity dispersion of the atoms with a precision (s.e.) of 53 p.p.m. during a single-line scan, taking ∼30 s. This is consistent with the sample s.d. (also 53 p.p.m.) over multiple scans, demonstrating the excellent reproducibility of the spectrometer. The measurement precision averages down to 3.7 p.p.m. after 200 scans. These values are ∼7 times better than the previous best results^28^,^29^,^30^, and also yields a near-two-fold reduction in the uncertainty of the excited-state hyperfine splitting in Cs^31^. Our measurement of the homogenous broadening component of the lineshape has a precision within a factor of two of the best ever measurements of natural linewidth. Modest improvements in the probe laser performance would deliver a new result for the excited state lifetime of Cs in a system that is experimentally and theoretically much simpler than that typically used for lifetime studies^32^,^33^.

## Results

### Transmission spectra

**Figure 1:**
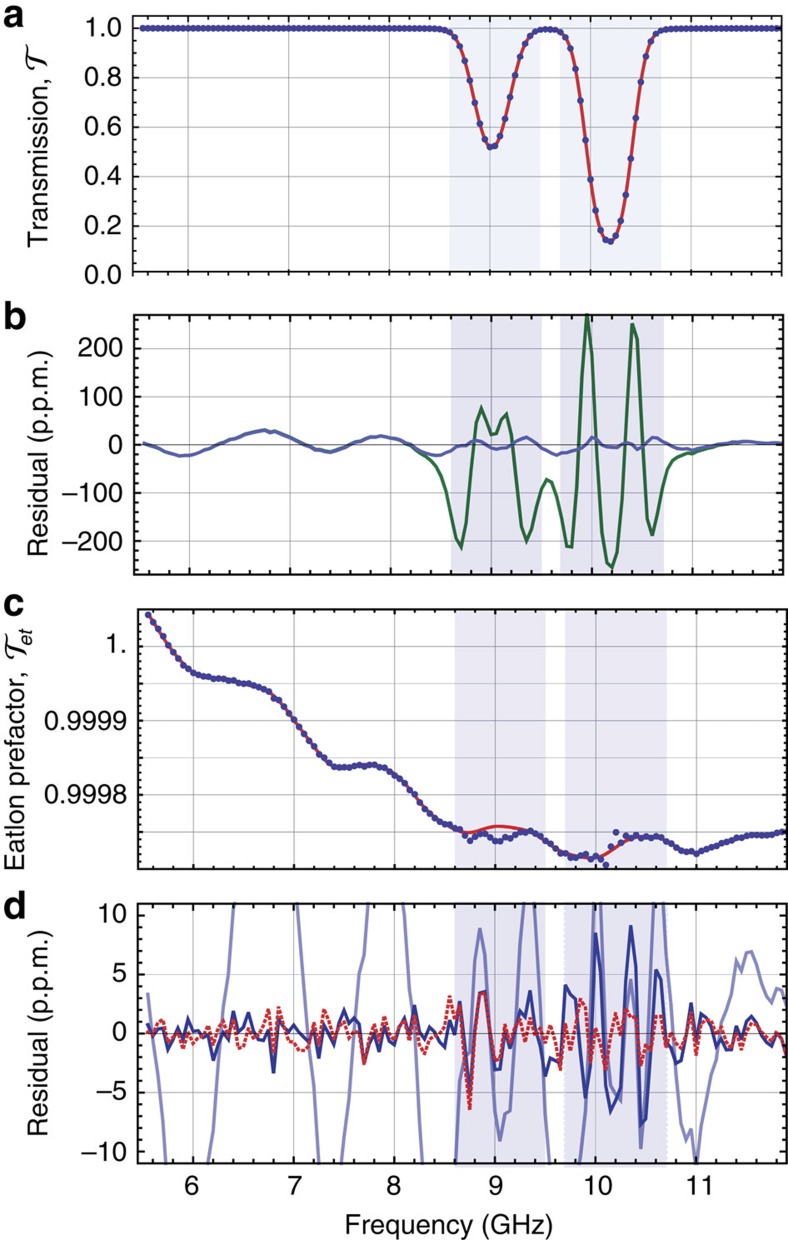
Typical transmission spectra and fit residuals. (**a**) Measured transmission at 296 K for the highest intensity, *I*/*I*_sat_=0.0028, averaged over 200 scans (points) and theory (solid). (**b**) Residuals including Voigt-only (dark green) and first-order correction (light blue). (**c**) Etalon pre-factor, equation (1), with 6 etalons (solid) and data divided by second-order power-corrected Voigt profiles (points), assuming Γ_*L*_=2.327 MHz. (**d**) Residuals after including first-order correction with 0 etalons (light blue) and 6 etalons (dark blue), and second-order with 6 etalons (dotted red).

We measure the transmission through the vapour cell as the probe laser frequency scans across the Cs D1 transition (6S_1/2_–6P_1/2_), shown in Fig. 1a. The relative noise in the measurement of the atomic transmission is set by the shot noise in the probe and reference beams, and provides 2 p.p.m. in a 1s measurement (at the highest optical powers used). The two absorption dips seen in Fig. 1a come from the hyperfine splitting in the excited state and the excellent signal-to-noise ratio enables us to determine this splitting to be *f*_HFS_=1167.716(3) MHz. The accuracy of this measurement exceeds that of the previous best determination^31^ in which a sub-Doppler atomic beam spectroscopy technique was used to measure *f*_HFS_. Spectroscopy in vapour cells is appealing for its ease of implementation, but as Gerginov *et al*. identified, such approaches often suffer from optical-pumping-related systematics that necessarily arise in the creation of sub-Doppler absorption features. Here, we avoid this by greatly improving the detection sensitivity and perturbatively accounting for non-linear absorption effects in our lineshape model.

The conventional description of an absorption lineshape, 
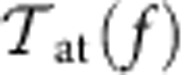
, of an atomic transition with a rest-frame transition frequency *f*_0_, is given by a convolution of a Lorentzian function with a natural (half) linewidth 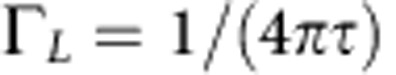 due to the finite lifetime 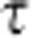 of the transition, with a Gaussian distribution having a *e*^*−*1^-width 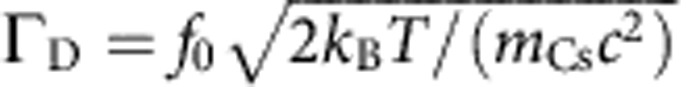 due to Doppler broadening of atoms of mass *m* at temperature *T*. By fitting a lineshape to the measured transmission data, with 
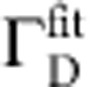
 as a fitting parameter, we extract the Doppler component, from which we infer the temperature. Systematic errors in 
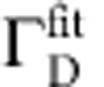
 are quantified by converting the independent PRT temperature measurements into an expected Doppler width, 
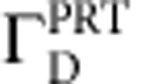
, using the CODATA values for *k*_B_ and *m*_Cs_, which we compare with 
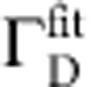
.

If the natural lineshape of the transition is a Lorentzian, then the resulting convolution is the well-known Voigt profile, which is commonly used to model dipole resonances. However, our extremely low-noise transmission measurements reveal deviations from the Voigt profile. Some of these deviations are technical in origin for example, instrumental broadening due to the lineshape of the probing laser, residual spontaneous emission from the probe laser, unwanted optical etalons and photodetector linearity; however, there is an important fundamental effect resulting from frequency-dependent optical pumping, which perturbs the atomic natural lineshape away from a Lorentzian. All of these effects, whether technical or fundamental, cause systematic perturbations to the lineshape, and must be taken into account to model the lineshape accurately.

### Least-squares fitting

**Figure 2:**
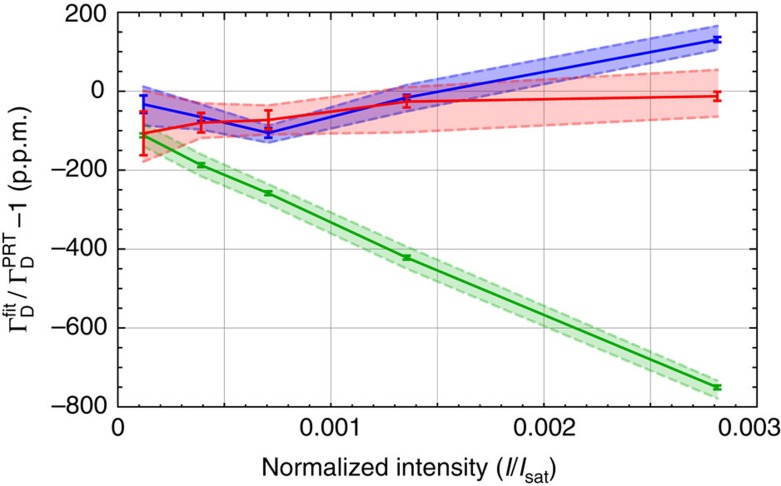
Power dependence of the fitted Doppler width. Fractional deviation between the fitted Doppler width and that inferred from PRT temperature are plotted for measurements at 296 K. Green points are fits using the conventional Voigt-only profile. Blue points are fits using the first-order intensity-dependent correction. Red points are fits using the second-order intensity-dependent correction. Solid curves are fits using our central estimate of 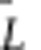
=2.327 MHz; dashed curves are for Γ_*L*_=2.320 MHz (upper) and Γ_*L*_= 2.334 MHz (lower) representing 
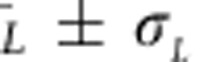
: the shaded regions indicate the sensitivity of 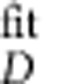
to Γ_*L*_ in this range. Error bars represent s.e. of fits to the average of 200 scans for fixed Γ_*L*_.

To demonstrate these deviations, we fit a model consisting of two Voigt profiles separated by *f*_HFS_, to the raw transmission data shown Fig. 1a. The residuals of this Voigt-only fit have characteristic `M'-shaped features near resonance, with amplitude ∼200 p.p.m. at the highest probe powers, as shown in the green trace in Fig. 1b. These features demonstrate the breakdown of the Voigt profile, indicating additional, unaccounted for physics, which causes a spurious linear dependence of 
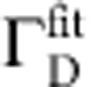
 on probe intensity, as shown in the green curve in Fig. 2. In what follows, we describe how we sequentially include additional physics in our transmission model to remove these, and other, systematic effects, leaving only the shot-noise limit of our detection apparatus.

In earlier work we showed that the `M'-shaped features in Fig. 1b arise from atomic population dynamics induced by the probe laser, which are significant even at exceedingly low intensities^25^. We subsequently calculated corrections to the Voigt profile up to linear order in the probe intensity^26^, to account for optical pumping. Including this first-order intensity-dependent corrections in our model suppresses the features substantially, as shown in the blue trace in Fig. 1b.

### Treatment of etalons

Far from the resonances, oscillations are evident in the residuals shown in Fig. 1b, which are clearly resolved due to our very high transmission measurement sensitivity (see also ref. 17). These oscillations arise from low-finesse etalons in the optics used to deliver the light to the atoms. An alternate thesis, that these features might arise from photoassociation of Cs atoms, can be discarded as these effects can be shown to be immeasurable at the present number density (see the Error Budget section of the Supplementary Discussion and ref. 34). Etalons are of particular concern for DBT since they introduce systematic errors in 
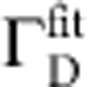
. In the Etalon Reduction section of the Supplementary Methods, we show that scattered light at the level of 0.1 p.p.b. of the beam power from non-reflecting surfaces can produce etalons with amplitude 30 p.p.m., consistent with the dominant etalon seen in Fig. 1b. Although obviously technical in nature, reducing the etalons beyond this already small amplitude is an experimental challenge. To handle these residual etalons in the analysis, we include etalons in our transmission model so that the total transmission is given by 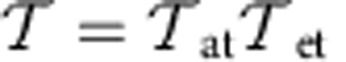, where 
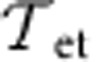
 includes *N*_et_ etalons and a slowly varying quadratic background:


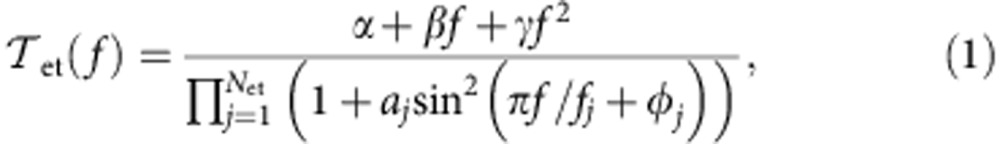


where *f*_*j*_ is the free-spectral range of etalon *j*.

We add etalons to the model until the residuals far from resonance are consistent with a white-noise background. For completeness, we can include up to *N*_et_=6 etalons, with the smallest resolved etalons having amplitude of a few p.p.m. The solid curve in Fig. 1c shows 
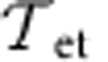
 for the highest power data; the points show the raw transmission data divided by the fitted 
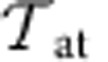
, demonstrating that 
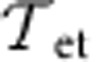
 is uncorrelated with the atomic transmission profile shown in Fig. 1a. The Etalon Reduction section of the Supplementary Methods has a detailed analysis of etalon fitting, showing that it is robust, and with *N*_et_≥2 etalons we recover a stable value of 
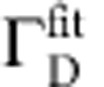
.

### Optical pumping perturbations

The dark-blue trace in Fig. 1d includes first-order power-dependent corrections, and 6 etalons. Away from resonance, the residuals are then consistent with the noise floor of our apparatus. However, around the deepest resonance we observe ∼10 p.p.m. features. To eliminate these we include second-order intensity-dependent corrections to the Voigt profile of each resonance (see the Lineshape Corrections at Second Order in Intensity section in the Supplementary Discussion):


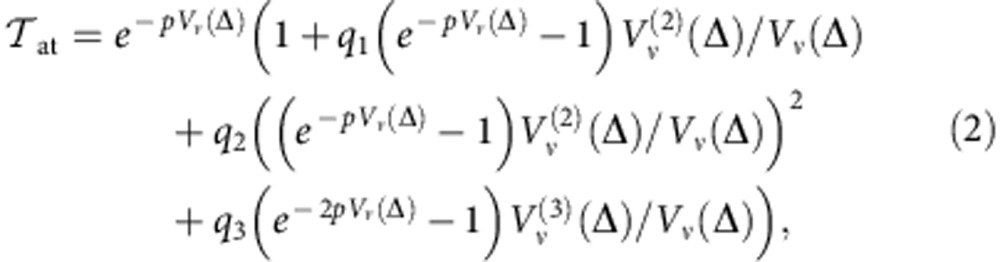


where *ν*=Γ_D_/Γ_*L*_, Δ is the detuning from the resonance, and the generalized Voigt profile is given by 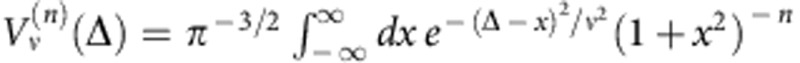, and the conventional Voigt profile is 
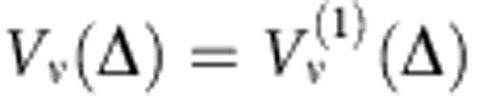
. For a cell of length *L* with linear absorption coefficient *α*, the optical depth on resonance is *p*=*αL*, *q*_1_∝*I* is the linear intensity-dependent coefficient^25^, and *q*_2,3_∝*I*^2^ are quadratic intensity-dependent coefficients.

The red-dotted trace in Fig. 1d shows the residuals after including second-order intensity corrections. The residuals are suppressed to ∼2 p.p.m., consistent with the detection noise floor, giving confidence that the transmission model accounts for systematic effects.

Figure 2 compares the fractional deviation between 
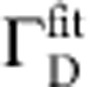
 and 
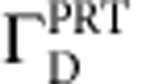
 as a function of incident intensity for the three possible models for the atomic lineshape: Voigt-only (green), first-order (blue) and second-order (red) intensity-dependent corrections. Naturally, the true Γ_D_ should be intensity independent. The simplest Voigt-only theory clearly exhibits a linear intensity dependence, leading to ∼750 p.p.m. discrepancy even at intensities as low as *I*/*I*_sat_=2.8 × 10^−3^ where *I*_sat_=2.5 mW cm^−2^ is the saturation intensity for the transition^35^. From ref. 26, we expect the first-order correction to have a residual quadratic dependence on intensity, which is consistent with the blue curve on Fig. 2. Finally, the second-order correction (red curve) is seen to suppress all intensity dependence to below the measurement precision. The simultaneous removal of all systematic features from the residuals together with the elimination of intensity dependence in the recovered 
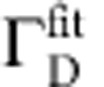
 gives a high degree of confidence that the model includes all relevant physics.

We note briefly that in fluorescence spectroscopy one should account for quantum interference between different hyperfine manifolds^36^,^37^,^38^,^39^,^40^. This is not an issue in absorption spectroscopy (see the Quantum Interference section of the Supplementary Discussion).

### Application to primary thermometry

In this section we demonstrate the power of our spectroscopic technique by applying it to the problem of primary thermometry, and determine a value for Boltzmann's constant using atomic spectroscopy.

The atomic vapour is thermalized at around *T*=296 K (that is, 
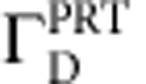
=215.123 MHz) by suspending it in a thermal isolator. The independent PRT measurement has an uncertainty of 0.55 mK (1.9 p.p.m.). We note the temperature drifted over the course of the experimental scans, such that 
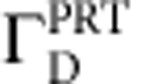
 is expected to vary from ∼214.96 to 215.13 MHz (see Supplementary Fig. 7).

After systematically removing the effects of optical pumping and etalons, the largest source of uncertainty in 
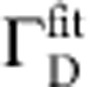
 comes from the uncertainty in Γ_*L*_. We obtain a value for Γ_*L*_ by directly fitting it to the data, which gives 
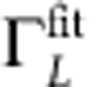
=2.327(7) MHz. This is consistent with, but also more precise than, an independent estimate, 
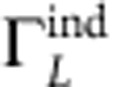
=2.331(19) MHz, given by the sum of the natural linewidth of the Cs 6*P*_1/2_ level, 
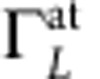
=2.287(6) MHz (ref. 35), and the laser linewidth, 
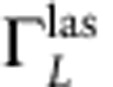
=0.044(18) MHz. From Fig. 2 we estimate that a 1kHz change in Γ_*L*_ leads to ∼5 p.p.m. change in 
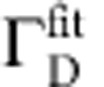
, so that the 6.5 kHz uncertainty in Γ_*L*_ contributes 32.5 p.p.m. uncertainty in 
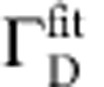
 (65 p.p.m. in *u*_*r*_(*k*_B_)). We note that if the probe linewidth were ∼1 kHz, then our measurement would yield a state-of-the-art value for the excited-state lifetime of Cs.

**Figure 3:**
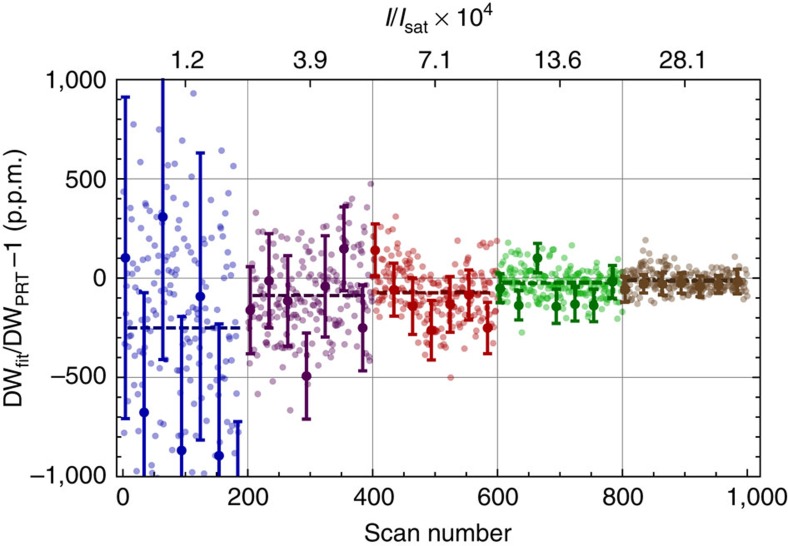
Scatter of Doppler width determinations. The deviation between 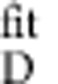 (using second-order intensity correction with Γ_*L*_=2.327 MHz) and 
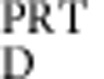
 are plotted, for each scan at 296 K. Colours and vertical gridlines demark different incident intensities (as shown along top axis), corresponding to normalized intensities in Fig. 2. Dashed lines are the mean of the corresponding set of scans, error bars are the estimated parameter error (±1*σ*) for each selected scan, and are consistent with sample s.d. Etalons are included in each fit, with parameters fixed by fits to the average of all scans within a power level, that is, etalon parameters are not free to vary within a particular power level.

We now briefly describe additional sources of uncertainty, which are quantified in Table 1 (see the Error Budget section of the Supplementary Discussion for a comprehensive description). Statistical error arising from the scatter in Fig. 3 contributes 2.9 p.p.m. to *u*_*r*_
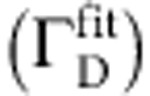
 (5.8 p.p.m. to *u*_*r*_(*k*_B_)). This is also consistent with the estimated s.e. for each point in Fig. 3, shown as error bars. The probe laser has a Gaussian noise component causing 0.88(39) MHz of broadening contributing 8 p.p.m. to *u*_*r*_
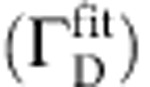
 (16 p.p.m. to *u*_*r*_(*k*_B_)). Residual effects of optical pumping after second-order power corrections contribute 7.5 p.p.m. to *u*_*r*_
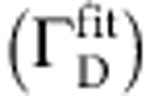
 (15 p.p.m. to *u*_*r*_(*k*_B_)). Misidentification of etalon parameters contributes 7.5 p.p.m. to *u*_*r*_
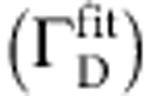
 (15 p.p.m. to *u*_*r*_(*k*_B_)). Possible unresolved etalons contribute 3 p.p.m. to *u*_*r*_(*k*_B_). Residual spontaneous emission from the laser, after filtering by the optical cavity, contributes 3.6 p.p.m. to *u*_*r*_(*k*_B_). In the Error Budget section of the Supplementary Discussion, we calculated the effects of atomic recoil caused by the probe beam itself and quantum interference between the two atomic resonances and found them to be negligible.

To determine a value for *k*_B_, we take a weighted mean of 
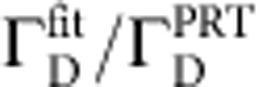
 extracted from fits using all the second-order corrected data shown on Fig. 2 (red points). We find *k*_B_=1.380545(98) × 10^−23 ^J K^−1^, where the 71 p.p.m. uncertainty is calculated in Table 1. This is consistent with the recommended CODATA value of 1.3806488(13) × 10^−23^ J K^−1^.

## Discussion

In conclusion, we have developed an atomic absorption spectrometer that operates with a transmission measurement precision of just 2 p.p.m. This has revealed several new phenomena. Combined with a perturbative treatment of optical pumping we are able to explain all of the observed effects to a level of precision never before demonstrated. The power of our technique is demonstrated by measuring the lineshape parameters of thermal vapour. The reproducibility is consistent with the quantum limits of measurement^41^ giving confidence that we have captured the relevant physics.

With this unprecedented sensitivity, we have measured the transmission spectrum of Cs at 895 nm at the laser intensity shot-noise limit. From these spectra, we derive a value for Boltzmann's constant with an uncertainty of 71 p.p.m., consistent with the recommended CODATA value, and the hyperfine frequency splitting of the 6^2^*P*_1/2_ level with the lowest uncertainty ever previously reported:


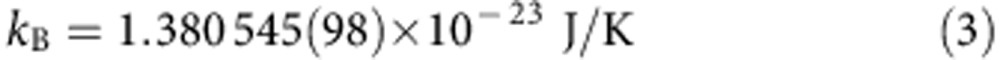



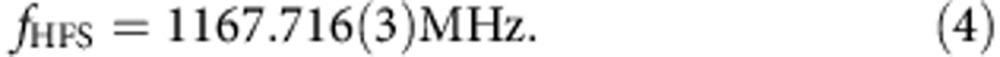


Statistical uncertainty contributes 6 p.p.m. to our error budget, after averaging spectra taken over a few hours of data acquisition. Our total uncertainty is dominated by imprecision in the literature value of the excited state lifetime of the Cs D1 transition (known to 0.26% (ref. 35)).

Although drifts in the experiment are likely in the apparatus on timescales required for multiple scans (for example, in the absolute optical frequency of the master laser), we average only the fitted parameters from each spectrum that are insensitive to the drifts; for example, the centre frequency of each resonance cannot be determined to a better accuracy than the stability of the master laser, but their difference can be averaged over long timescales to achieve a lower uncertainty.

A future experiment designed to further minimize etalons, and using a higher power, sub-kHz linewidth probe laser with greater tuning range (∼30 GHz) would eliminate probe laser effects, allow better estimation of etalon contamination, and lower the contribution of optical pumping. This opens the door to the best measurement of the Cs D1 transition lifetime, and a p.p.m.-level measurement of *k*_B_ thus making an important contribution to efforts to redefine the kelvin.

## Methods

### Probe light preparation

**Figure 4:**
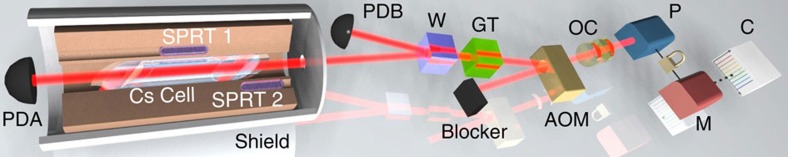
High-accuracy linear absorption spectrometer for probing the D1 transitions in atomic Cs vapour. A probe laser (P) is offset-frequency locked to a master laser (M), which is in turn compared against a stabilized optical frequency comb (C). The probe laser output is spectrally and spatially filtered with an optical cavity (OC) before being deflected by an acouto-optic modulator (AOM). The beam is then passed through a Glan-Taylor (GT) prism and divided on a Wollaston (W) beam splitter. The power of the beam transmitted through the Cs cell inside the thermal and magnetic shield is measured on a photodetector PDA and compared with a reference beam measured on photodetector PDB. SPRT1 and SPRT2 are standard platinum resistance thermometers from which we independently measure the cell temperature.

The spectrometer is pictured in Fig. 4a. The probe laser (P) is tightly locked at a tunable frequency difference away from a master laser (M), which is itself stabilized to an atomic transition in Cs. The frequency stability of the master laser is compared with a reference frequency provided by an optical frequency comb (C). The probe laser is spectrally and spatially filtered using a combination of an optical cavity (OC) of moderate finesse 
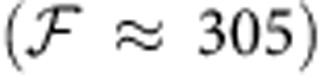
 and single-mode fibre (not shown). This reduces the spontaneous emission content of the probe beam from 1.6% to below 0.01%. The optical power is actively stabilized with an acousto-optic modulator (AOM). It is then delivered into a vacuum chamber in which the vapour cell is mounted in a thermal and magnetic shield. Calibrated standard platinum resistance thermometers (SPRTs) were used to independently measure the temperature of the shield and control it with a precision of a few millikelvin. Spatial temperature gradients are suppressed to the same level.

### Measurement scheme

Inside the vacuum chamber, the probe light is split into two beams using a combination of a Glan–Taylor polarizing prism (GT) and Wollaston beam splitter (W). One beam passes through a Cs cell embedded inside the thermal and magnetic shield and is measured by photodiode A (PD A); the other beam is measured directly by photodiode B (PD B). The ratio of these photodiode signals gives us the transmission ratio, 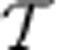, which was stable to better than 10^−6^. The incident power is actively controlled using the level on PD A while the frequency of the incident light is set with an absolute uncertainty of 2 kHz, limited by our ability to realize the unperturbed transition frequency of the Cs saturated absorption feature to which the master laser was locked. Precision in the radio frequency (RF) frequency detuning between the master and probe lasers was ensured by referencing the offset-frequency locking electronics to a Cs beam clock-disciplined maser. Technical details about the apparatus are given in the Supplementary Methods section.

## Additional information

**How to cite this article:** Truong, G.-W. *et al*. Accurate lineshape spectroscopy and the Boltzmann constant. *Nat. Commun.* 6:8345 doi: 10.1038/ncomms9345 (2015).

## Supplementary Material

Supplementary Information Supplementary Figures 1-11, Supplementary Discussion, Supplementary Methods and Supplementary References

## Figures and Tables

**Table 1:** Error budget for the determination of the Boltzmann constant at 296 K.

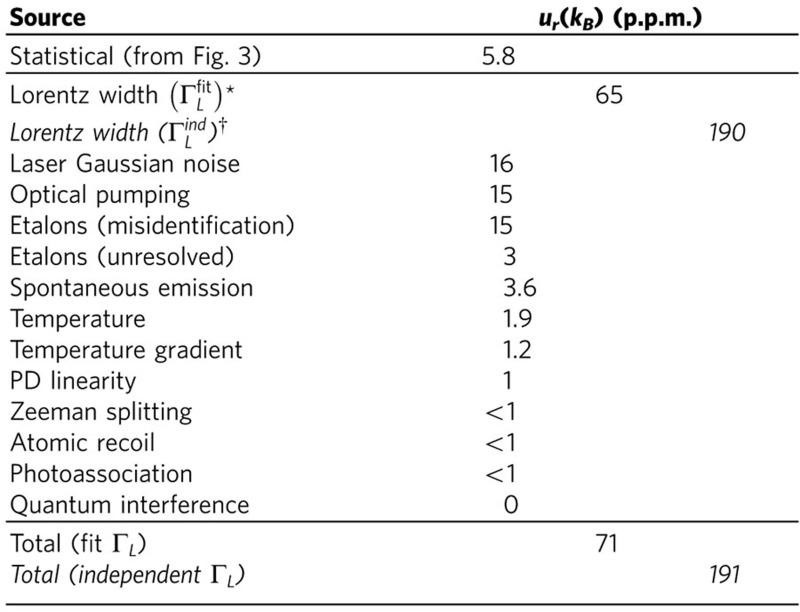

Optical pumping shifts are reported at *I*/*I*_*sat*_=3 × 10^−3^ assuming second-order corrections to the Voigt profile. *u_r_* denotes fractional uncertainty.

^*^Estimated from fits to scan data with free Lorentz width.

^†^Estimated from published uncertainty in Cs lifetime, plus uncertainty in the independent estimate of the laser linewidth.
