# Supplementary material for: Accurate lineshape spectroscopy and the Boltzmann constant
Source: Nat Commun. 2015 Oct 14;6:8345. doi: 10.1038/ncomms9345 (PMC4633988; doi:10.1038/ncomms9345)
Supplement: Supplementary Information — Supplementary Figures 1-11, Supplementary Discussion, Supplementary Methods and Supplementary References [file ncomms9345-s1.pdf]

SUPPLEMENTARY FIGURES

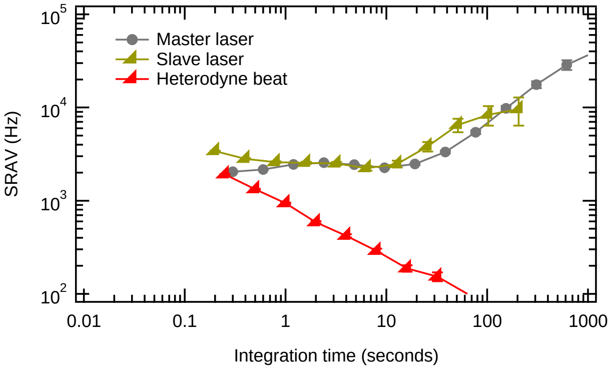

Supplementary Figure 1. Square-root-Allan-variance (SRAV) of the absolute frequency stabilities of the master laser (grey circles), slave laser (olive triangles) and the heterodyne beat (red triangles).

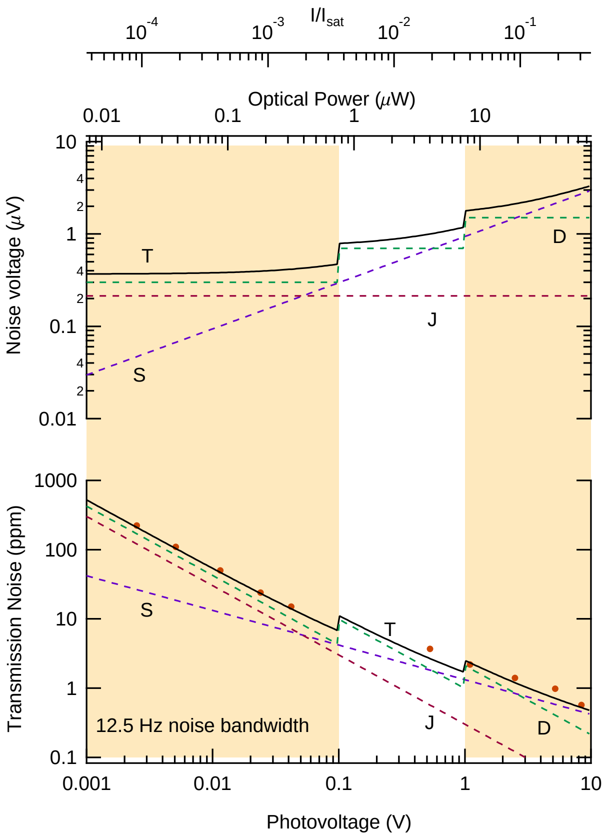

Supplementary Figure 2. Top: Calculated shot noise (S) and Johnson noise (J) voltages as a function of the photosignal. The DMM (D) input noise was empirically determined and found to depend on the measurement range setting (indicated by the shaded regions). Bottom: Measured noise in the atomic transmission channel (circles) compared to the calculated (with no free parameters) contributions from the DMM, Johnson noise, shot noise and total (T) are also shown for comparison.

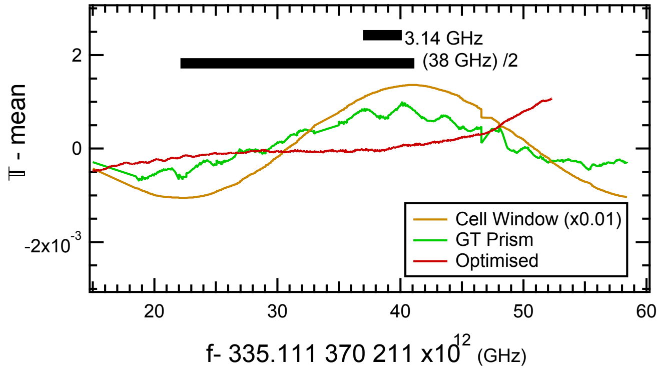

Supplementary Figure 3. The probe laser was scanned over a wide frequency range to search for etalons in the optical layout.

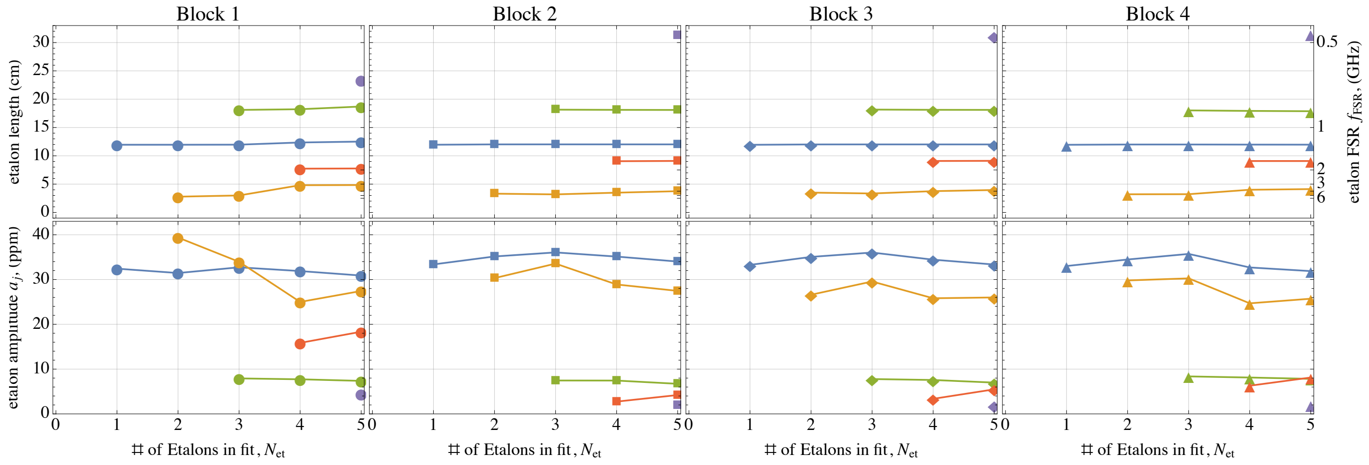

Supplementary Figure 4. Etalon parameters from fitting to averages of blocks of 50 scans. For each block, $N_{\rm et} = 1, ..., 5$ etalons are included in the fitting function, from which we extract free-spectral ranges (top row), amplitudes (bottom row) and phases (not shown) for each of the $j \in \{1, ..., N_{\rm et}\}$ etalons. All etalon parameters are left free, so etalon parameters can in principle vary with $N_{\rm et}$. Importantly, the etalon FSRs (expressed in terms of the equivalent resonant-cavity length $L = c/(2f_{\rm FSR})$ are quite stable as more etalons are added.

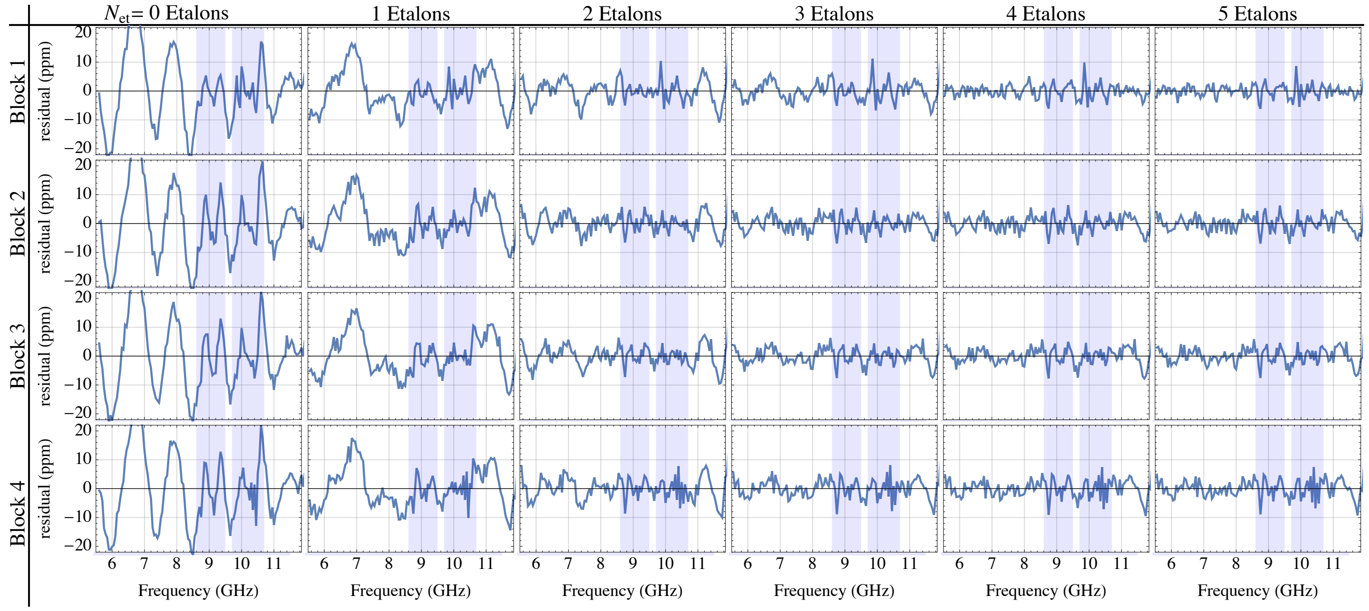

Supplementary Figure 5. Residuals after fitting with increasing number of etalon terms showing systematic removal of etalon contributions. The 200 scans in the highest power data set were blocked into groups of 20 scans and averaged. For each block, a series of fits are performed with sequentially more etalon terms added to the fitting function, starting at zero etalons. The residuals are plotted here for each block, and after 0, ..., 5 etalons are added to the fitting. Shaded bands indicate the location of the resonances.

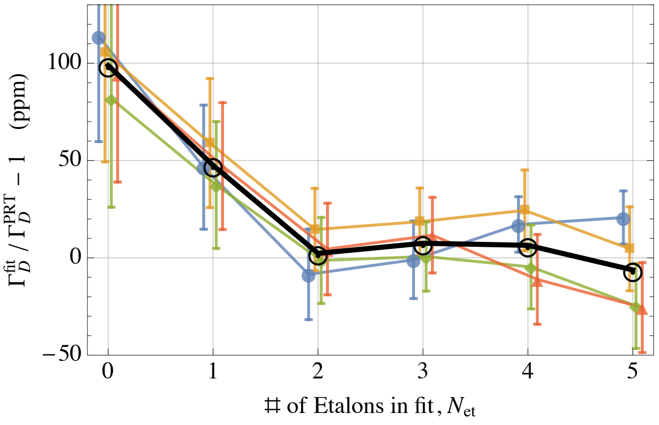

Supplementary Figure 6. Dependence of $\Gamma_D^{\rm fit}$ on number of fitted etalons. Coloured lines correspond to different blocks (Block 1: blue circles; Block 2: orange squares; Block 3: green diamonds; Block 4: red triangles). Hollow circles are the mean of the block values. Error bars are standard errors reported by the fitting. After 2 etalons are included in the fit, additional etalons do not significantly change the value of $\Gamma_D^{\rm fit}$. Note that we have offset each data series along the horizontal axis by a small amount to make error bars visible.

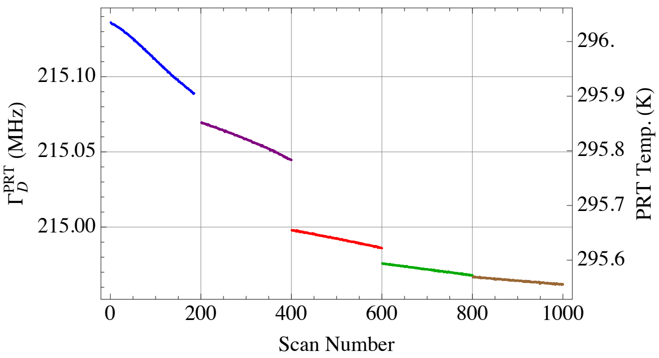

Supplementary Figure 7. $\Gamma_D^{\rm PRT}$ vs. scan number. Colours indicate different sets of scans at different power levels; low power at left, high power at right, in correspondence with Fig. 3 of the main text.

SUPPLEMENTARY DISCUSSION

Error Budget

Here we give a detailed description and derivation of the uncertainties reported in Table I of the main text. $u_r(X)$ denotes the fractional uncertainty in X.

Statistical: For each power level, the statistical contribution is computed by fixing the etalon parameters (to values obtained from fits to coaveraged scans, see below), and extracting $\Gamma_D^{\rm fit}$ from each scan independently. These are shown in Supplementary Figure 8, and we find a statistical error of 2.9 ppm in $\Gamma_D^{\rm fit}$, [which represents 5.8 ppm in $u_r(k_{\rm B})$].

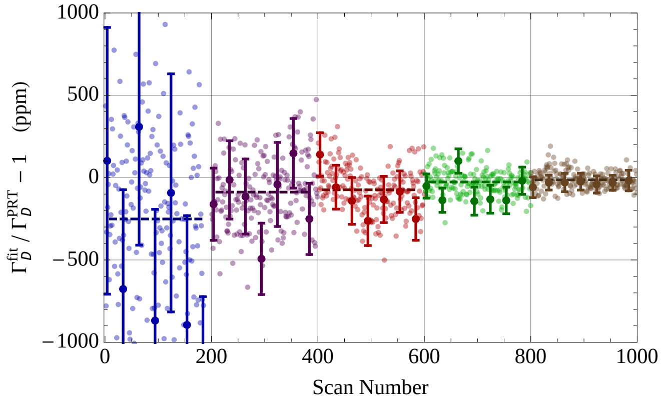

Supplementary Figure 8. Deviation between $\Gamma_D^{\rm fit}$ (using second-order Voigt correction) and $\Gamma_D^{\rm PRT}$, for each scan at 296 K, with $\Gamma_L = 2.327$ MHz. Colours and vertical lines demark different incident intensities (lowest at left, corresponding to normalised intensities in main text). Dashed lines are the mean of the corresponding set of scans, error bars are the estimated parameter error ($\pm 1\sigma$) for each selected scan, and are consistent with sample standard deviation.

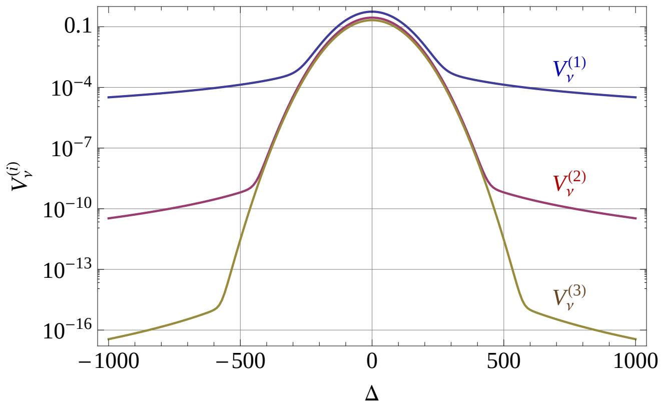

Supplementary Figure 9. Generalised Voigt profiles versus detuning, $\nu = 100$.

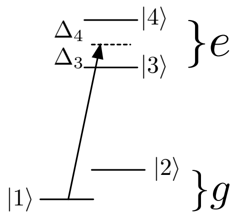

Supplementary Figure 10. Toy model of an atom with two non-degenerate excited states.

Lorentz Width: Our probe laser has a Lorentzian linewidth, which adds to the atomic linewidth, $\Gamma_L = \Gamma_L^{\rm at} + \Gamma_L^{\rm las}$. To determine Γ_L, there are two alternatives: either to treat Γ_L as a free fitting parameter (together

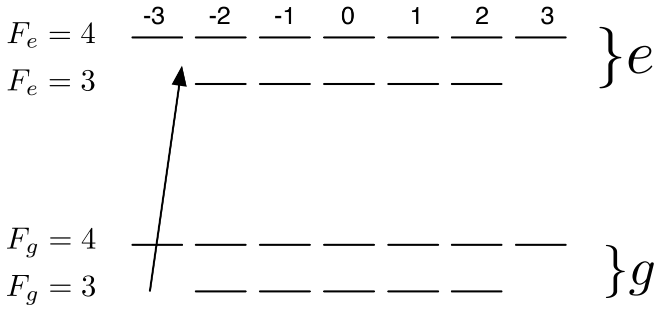

Supplementary Figure 11. D_1 transition showing ground and excited hyperfine manifolds.

with Γ_D), or to fix $\Gamma_L^{\rm at}$ to the best reported literature value and independently estimate $\Gamma_L^{\rm las}$.

In the former route we fit to the average of 200 independent scans for each probe power level we retrieve a value of $\Gamma_L^{\rm fit} = 2.327(7)\,$MHz. In the latter route, we take $\Gamma_L^{\rm at} = 2.287(6)$ MHz for the natural linewidth of the Cs $6P_{1/2}$ level [3], and an independent estimate of the laser noise $\Gamma_L^{\rm las} = 0.044(18)$ MHz (see above), which totals $\Gamma_L^{\rm ind} = 2.331(19)$ MHz. These two estimates for Γ_L are consistent within their measurement uncertainty, and so we take the value with smaller uncertainty $\Gamma_L = 2.327(7)\,$MHz. In particular, the solid curves in Fig. 2 of the main text assume the central value $\Gamma_L = 2.327\,$MHz, and the dashed curves correspond to the upper and lower bounds of the uncerainty range, $\Gamma_L = 2.320\,$MHz and $2.334\,$MHz.

We note that the relative precision in our measurement of Γ_L is within a factor of two of the precision of the best measurements of the Cs D1 transition lifetime, $\tau_{\rm Cs}$ [8, 9], and matches the precision of the best synthesised value of $\tau_{\rm Cs}$ [3] formed by combining these measurements.

We also see from Fig. 2 of the main text that a 1 kHz variation in Γ_L leads to ~ 5 ppm change in $\Gamma_D^{\rm fit}$, which is consistent with a 1:1 tradeoff between increases in Γ_L and decreases in $\Gamma_D^{\rm fit}$, when $\Gamma_D \approx 215$ MHz. This implies that our ± 6.5 kHz uncertainty in $\Gamma_L^{\rm fit}$ contributes 32.5 ppm to $u_r(\Gamma_D^{\rm fit})$ (equivalent to 65 ppm to $u_r(k_{\rm B})$). The $\pm$19kHz uncertainty in $\Gamma_L^{\rm ind}$ represents 190 ppm in $u_r(k_{\rm B})$.

Laser Gaussian Noise: The probe-laser spectrum has a Gaussian contribution [1]. This was measured and found to have a width of 0.88(39) MHz. The Gaussian component sums in quadrature with Γ_D, and so contributes 8 ppm to $u_r(\Gamma_D^{\rm fit})$ [16 ppm to $u_r(k_{\rm B})$].

Optical Pumping: The second-order power-dependent corrections suppress the fit residuals in (shown in Fig. 2(d) of the main text) to the measurement noise floor, so there is no statistically significant signature of optical pumping. Nevertheless, any residual intensity dependence in the second-order intensity-corrected $\Gamma_D^{\rm fit}$ is expected to scale as $(I/I_{\rm sat})^3$. We therefore estimate the error arising from uncorrected third-order optical pumping by fitting a cubic through the highest intensity red data points in Fig. 2 of the main text. This gives a maximum deviation of 15 ppm in $u_r(\Gamma_D^{\rm fit})$ at the *maximum* probe intensity, and smaller contributions at lower intensities. Forming the weighted average over all intensities in Fig. 2 of the main text contributes 7.5 ppm to $u_r(\Gamma_D^{\rm fit})$, [15 ppm in $u_r(k_{\rm B})$].

Etalons: Etalons enter into the uncertainty budget in two ways. Firstly, etalon parameters may be misidentified during fitting, and so contaminate $\Gamma_D^{\rm fit}$. Secondly unresolved etalons masked by measurement noise may introduce residual systematic shifts to Γ_D.

In Fig. 2 of the main text we fit $\Gamma_D^{\rm fit}$ with free etalon parameters. The weighted average of the standard errors is 8 ppm in $u_r(\Gamma_D^{\rm fit})$, which is a convolution of the statistical noise discussed above and uncertainties arising from possible misidentification of the etalon parameters during fits. When the statistical errors in $\Gamma_D^{\rm fit}$ are deconvolved from the standard errors in Fig. 2 of the main text, we find the systematic error due to free etalon parameters in the fitting routine to be $(8^2 - 2.9^2)^{1/2} = 7.5$ ppm in $u_r(\Gamma_D^{\rm fit})$, [15 ppm in $u_r(k_{\rm B})$]. As a check, this figure is consistent with the errors in Supplementary Figure 6, which imply $u_r(\Gamma_D^{\rm fit}) = 9$ ppm for the hollow circles (i.e. averaging over the fits to each of the four blocks of 50 scans).

We bound the error arising from unresolved etalons in two ways: by synthesising data with false etalons, and by computing the fractional shift in Γ_D after the addition of the $j^{\rm th}$ etalon in the fit function. Both approaches give worst-case shifts in $\Gamma_D^{\rm fit}$ that are comparable to the amplitude of the etalon. This is also borne out by comparing the variation of $\Gamma_D^{\rm fit}$ shown in Supplementary Figure 6 as etalons are added with amplitudes given by the corresponding values in Supplementary Figure 4(bottom). The largest unresolved etalons are smaller than the measurement noise floor, so these cannot contribute more than 3 ppm to $u_r(k_{\rm B})$.

Atomic recoil: We have already previously discussed the perturbation of the atomic gas by the probe beam due to optical pumping, which modifies the measured lineshape by changing the atomic energy level occupancies away from the thermal equilibrium condition. The probe beam may, by imparting a recoil momentum after atomic absorption of a probe photon, directly modify the velocity distribution. This would not be a problem if: I) the recoil directions of the atoms were isotropic (which it cannot be since the momentum of every probe beam photon is in same the direction), and no atom that has suffered a change in momentum is not remeasured by the probe beam.

We provide a worst-case estimate of the perturbation to the measured Doppler width by assuming that after every absorption event, the re-emitted photon is propagating back towards the probe laser. In this case, the

recoil velocity of the atom is 6.7 mm/s in the same direction as the propagation of the probe beam, causing a Doppler shift of $\Delta_{\rm recoil}$ =7.8 kHz. The observed distribution of atomic velocities (along the beam axis) is then composed of a sum of Gaussians shifted by an amount $(q-1)\nu_{\rm recoil}$ weighted according to the probability p_q of absorbing q photons before exiting the beam volume, i.e.

$$\sum_{q=1}^{\infty} p_q \exp\left[-\left(\frac{f-(q-1)\Delta_{\rm recoil}}{\Gamma_D}\right)^2\right] \quad (1)$$

We assume that the probability of absorbing q photons is drawn from a Poisson distribution of mean μ. The mean number of photons absorbed by any atom in the beam is given by the ratio:

$$\mu = \frac{p_\gamma \Phi_{\rm photons}}{\nu^{-1}\Phi_{\rm atoms}}. \quad (2)$$

The numerator is the effective photon flux that may undergo absorption. This consists of a photon flux term $\Phi_{\rm photons} = P\lambda/(hc) \approx 2.70\times10^{12}$ (using $P \approx 600$ nW which is the highest optical probe power used, $\lambda \approx 895$ nm is the resonance wavelength and h is the Planck constant), and the probability of absorption when the photon frequency is tuned to resonance p_γ. We will take $p_\gamma = 1$ as a worst-case approximation. The denominator is the effective atomic flux through the probe beam that may undergo absorption. The atomic flux is given by $\Phi_{\rm atoms} = \rho V/\tau_{\rm T} \approx 3.08\times10^{15}$, where the number density is $\rho = N_A U/(RT) \approx 7.1\times10^{16}$ atoms/m^3 (using the vapor pressure $U \approx 2.7\times10^{-4}$ torr at a temperature of $T = 273$ K) N_A and R are the Avogadro and Universal Gas constants respectively. $V \approx 2.4\times10^{-7}$ m^3 is the volume of beam of radius 1 mm and length 75 mm. $\tau_{\rm T} = 5.4\,\mu$s is the most probable time to transit a distance of one beam radius.The ratio $\nu^{-1} = \Gamma_L/\Gamma_D = 0.011$ accounts for the approximate fraction of atoms whose resonant frequency is within an atomic linewidth away from the probe laser frequency.

Under these worst-case approximations, $\mu \approx 0.081$; the probability of an atom interacting with zero, one and two photons as it transits the beam is 0.92, 0.075 and 0.0031, respectively. The probability of interacting with more photons is less than 10^{-4}. After fitting a simulated absorption profile (consisting of a sum of shifted Gaussian absorption profiles in accordance with Supplementary Equation 1) to a single Gaussian profile, the retrieved Doppler width Γ_D is perturbed by no more than 2 parts in 10^{10}. The deposition of momentum into the atomic ensemble by the probe laser is completely negligible in this case when the probe beam power is so low.

Photoassociation and diffuse bands: In dense atomic gases, additional broad spectral features extending over 100 nm appear in the wings of the absorption lines[10]. Studies using number densities exceeding one million times greater than those in our experiment have empirically determined that the absorption coefficient at red detunings $\Delta\lambda$ between 1 nm to 170 nm of the Cs D1 line have the following functional dependence:

$$\alpha = 9.46\times10^{-32} P(\delta)\frac{N^2}{(\Delta\lambda)^2} \quad (3)$$

where N is the number density and the function P is defined:

$$P(\delta) = 1.0317 + 2.2943\delta - 0.6319\delta^2 + 0.0732\delta^3, \quad (4)$$

with $\delta = \Delta\lambda/42.6$nm being the detuning normalised to the fine structure splitting. The N^2 dependence of the broadband absorption reduces this effect to unobservable levels in our experiment ($\alpha \ll 10^{-9}$).

Lineshape Corrections at Second Order in Intensity

Here we derive Eq. 2 of the main text, which gives the second-order intensity-dependent correction to the Voigt profile. We follow closely the derivation in [11].

We begin by computing the spectral dependence on the atomic populations for a three level atom, consisting of two ground states: one optically active, $|1\rangle$, and the other optically inactive, $|2\rangle$, and an excited state $|3\rangle$. Transitions between states $|1\rangle$ and $|3\rangle$ are optically driven, and state $|3\rangle$ can relax to either of the ground states. The population rate equations are [11]

$$\dot{\mathbf{P}} = M\mathbf{P}, \quad M = \begin{pmatrix} \frac{\Omega^2 f(t)/2}{1+\Delta^2} & 0 & 2\beta + \frac{\Omega^2 f(t)/2}{1+\Delta^2} \\ 0 & 0 & 2(1-\beta) \\ -\frac{\Omega^2 f(t)/2}{1+\Delta^2} & 0 & -2-\frac{\Omega^2 f(t)/2}{1+\Delta^2} \end{pmatrix}, \quad (5)$$

where $\mathbf{P} = \{P_1, P_2, P_3\}$, Δ is the detuning between the laser frequency and the atomic transition, β is the branching ratio from state $|3\rangle$ to $|1\rangle$, Ω is the peak atomic Rabi frequency (proportional to the electric field amplitude) and $\sqrt{f(t)}$ represents the temporal profile of the Rabi frequency experienced by the atom as it moves through regions of different intensity. We write the Rabi frequency in this form since in what follows we will expand the atomic response in powers of $\Omega^2/(1+\Delta^2)$. For simplicity of presentation, we will assume that the equilibrium atomic level populations are $\mathbf{P}_{\rm eq} = \{1/2, 1/2, 0\}$, i.e. we ignore degeneracies in the ground state manifold. Accounting for degeneracies is a simple modification to the relative fractions for P_1 and P_2 at equilibrium.

The light is absorbed as it propagates through the atomic medium. While Supplementary Equation 5 is defined with respect to the rest-frame of a particular atom, the axial evolution of the field in the lab-frame is governed by [11]

$$\frac{\partial \ln\Omega^2}{\partial z} = 2\,\pi^{3/2}K\int_{-\infty}^{\infty} d\Delta_{v_z}\frac{e^{-(\Delta-\Delta_{v_z})^2/\nu^2}}{\pi^{3/2}(1+\Delta_{v_z}^2)}P(\Delta_{v_z}), \quad (6)$$

where $P = P_3 - P_1$, $K = \rho_0\mu^2/(\sqrt{\pi}\varepsilon_0\hbar v_0)$, μ is the transition dipole moment and $v_0 = \sqrt{2k_BT/m}$ is the mean thermal atomic velocity for an atom of mass m. The notation Δ_{v_z} indicates that a given atom is subject to a detuning which depends on its axial velocity via the Doppler shift. If the field intensity is weak, so that on resonance populations are negligibly perturbed from thermal equilibrium, then $P = -1/2$, and the integral yields $\Omega^2(z) = \Omega_0^2 e^{-\pi^{-3/2}KzV_\nu(\Delta)}$. Since $\mathcal{T}(z) = \Omega^2(z)/\Omega_0^2$, we recover $\mathcal{T} = e^{-\pi^{-3/2}KzV_\nu(\Delta)}$, which is the conventional Beer's law result for a Voigt atomic lineshape.

For larger Ω^2, perturbations to P become significant, and we need to compute corrections to P arising from the dynamics described by Supplementary Equation 5. Since we are concerned with small but significant pump induced perturbations to the thermal population, we do a perturbative analysis of Supplementary Equation 5 around the limiting case where $\Omega = 0$.

By inspection of Supplementary Equation 5 we see that Ω^2 only ever appears as part of the ratio $\Omega^2/(1+\Delta^2)$, so it must appear in the same ratio in P, which expand in powers of Ω^2 [11]:

$$P(\Delta) = a_0 + a_1\frac{\Omega^2}{1+\Delta^2} + a_2\left(\frac{\Omega^2}{1+\Delta^2}\right)^2 + O(\Omega^6), \quad (7)$$

where $a_0 = -1/2$ and other a_j are expansion coefficients, which contain all the complexity of the ballistic atomic motion across the beam, including averages over transverse velocity classes and trajectories across the beam. Here P is defined in the lab-frame, so implicitly depends on the axial and radial position (z, r).

Substituting Supplementary Equation 7 into the LHS of Supplementary Equation 6, and evaluating the integral over Δ_{v_z} yields

$$\frac{\partial \ln\Omega^2}{\partial z} = 2\,\pi^{3/2}K\sum_{j=0}^{2} a_j V_\nu^{(j+1)}(\Delta)\,(\varepsilon\,\Omega)^2 + O(\Omega^6), \quad (8)$$

where we have introduced a pseudo-parameter $\varepsilon \equiv 1$ which we will use later to find a power-series solution, $\nu = \Gamma_D/\Gamma_L$, and we define a generalised Voigt profile

$$V_\nu^{(n)}(\Delta) = \int_{-\infty}^{\infty} d\Delta_{v_z}\,\frac{e^{-(\Delta-\Delta_{v_z})^2/\nu^2}}{\pi^{3/2}(1+\Delta_{v_z}^2)^n}$$

so that the conventional Voigt profile is

$$V_\nu(\Delta) \equiv V_\nu^{(1)}(\Delta) = \mathrm{Re}\{e^{-(\Delta+i)^2/\nu^2}\mathrm{erfc}[(1-i\Delta)/\nu]/\sqrt{\pi}\}.$$

$V_\nu^{(2)}$ may be evaluated by noting that

$$\frac{1}{(1+x^2)^2} = \lim_{\xi\to1}\left\{\frac{\xi^2}{(\xi^2-1)\,(\xi^2x^2+1)} + \frac{1}{(1-\xi^2)\,(x^2+1)}\right\}.$$

Convolving this sum of two Lorentzians with a Gaussian thus yields a sum of Voigt functions,

$$\begin{aligned} V_\nu^{(2)}(\Delta) &= \lim_{\xi\to1}\left\{\frac{\xi^2}{(\xi^2-1)}V_{\xi\,\nu}(\xi\,\Delta) + \frac{1}{(1-\xi^2)}V_\nu(\Delta)\right\} \\ &= \mathrm{Re}\{\frac{\nu + \sqrt{\pi}e^{-\frac{(\Delta+i)^2}{\nu^2}}\left(i\Delta+\nu^2/2-1\right)\mathrm{erfc}[\frac{1-i\Delta}{\nu}]}{\pi\nu^3}\}. \end{aligned}$$

Higher order correction terms can be calculated iteratively, using the same method. In particular

$$V_\nu^{(3)}(\Delta) = \mathrm{Re}\left\{\left(2\nu\,(2i\,\Delta+3\,\nu^2-2) + \sqrt{\pi}e^{-(\Delta+i)^2/\nu^2}\mathrm{erfc}\big[(1-i\Delta)/\nu\big](2(-2+3i\,\Delta)\nu^2 - 4(\Delta+i)^2+3\nu^4)\right)/(8\pi\nu^4)\right\}.$$

These are shown in Supplementary Figure 9.

Supplementary Equation (8) can be solved analytically for $\Omega^2(z)$ (for any order of expansion), however it becomes cumbersome. Instead, we expand in powers of ε, by substituting $\Omega^2(z,r) = \Omega_0^2(z,r) + \varepsilon\,\Omega_1^2(z,r) + \varepsilon^2\Omega_2^2(z,r) + ...$ into Supplementary Equation 8, and solving order-by-order in powers of ε, with initial conditions $\Omega^2(0,r) = \Omega_0^2(r)$ (where the radial dependence is parametric). Physically, this is simply an expansion around the zero-power limit. We find

$$\Omega_0^2(z,r) = \Omega_0^2(r)e^{-pV_\nu(\Delta)}, \quad (9)$$

$$\Omega_1^2(z,r) = \Omega_0^4(r)\frac{a_1e^{-pV_\nu(\Delta)}(e^{-pV_\nu(\Delta)}-1)V_\nu^{(2)}(\Delta)}{a_0V_\nu(\Delta)}, \quad (10)$$

$$\Omega_2^2(z,r) = \Omega_0^6(r)\left(\frac{a_1^2e^{-pV_\nu(\Delta)}(e^{-pV_\nu(\Delta)}-1)^2V_\nu^{(2)}(\Delta)^2}{a_0^2V_\nu(\Delta)^2} + \frac{a_2e^{-pV_\nu(\Delta)}(e^{-2pV_\nu(\Delta)}-1)V_\nu^{(3)}(\Delta)}{2a_0V_\nu(\Delta)}\right), \quad (11)$$

where $p = -2\,\pi^{3/2}K\,a_0z > 0$.

Since the intensity is proportional to $\Omega^2(z,r)$, we compute the transmission $z = L$ as

$$\mathcal{T} = \frac{\int_0^\infty r\,dr\,\Omega^2(L,r)}{\int_0^\infty r\,dr\,\Omega_0^2(r)}. \quad (12)$$

This gives Eq. (2) of the main text:

$$\begin{aligned}\mathcal{T} = e^{-pV_\nu(\Delta)}\Big(1 &+ q_1(e^{-pV_\nu(\Delta)}-1)V_\nu^{(2)}(\Delta)/V_\nu(\Delta)\\ &+ q_2\big((e^{-pV_\nu(\Delta)}-1)V_\nu^{(2)}(\Delta)/V_\nu(\Delta)\big)^2\\ &+ q_3(e^{-2pV(\Delta,\nu)}-1)V_\nu^{(3)}(\Delta)/V_\nu(\Delta)\Big),\end{aligned}$$

where $q_1 = (a_1\overline{\Omega_0^4})/(a_0\overline{\Omega_0^2})$, $q_2 = (a_1^2\overline{\Omega_0^6})/(a_0^2\overline{\Omega_0^2})$ and $q_3 = (a_2\overline{\Omega_0^6})/(2a_0\overline{\Omega_0^2})$, and $\overline{\Omega_0^n} = \int_0^\infty r\,dr\,\Omega_0^n(r)$.

It follows that q_1 is proportional to the intensity, whilst q_2 and q_3 are proportional to the square of the intensity. This is made explicit if we assume that the input probe beam has a gaussian transverse profile at $z = 0$, $\Omega_0^2(r) = 4\Omega_0^2 e^{-2(r/r_0)^2}$, and we find $q_1 = 2\,a_1\Omega_0^2/a_0$, $q_2 = 16a_1^2\Omega_0^4/(3a_0^2)$ and $q_3 = 8a_2\Omega_0^4/(3a_0)$. In particular, $q_2 = 4q_1^2/3$, so that q_2 is determined by q_1. Since we do not know the parameters $a_{1,2}$, or the optical depth we treat p and q_i as fitting parameters for each resonance.

Quantum Interference

The splitting between the hyperfine manifolds of $\delta \approx 1.17$ GHz is around 300 times larger than the natural linewidth of each transition, $\Gamma \approx 4.57$ MHz (FWHM). Thus, at a detuning half-way between the resonances, the detuning is around 150 natural linewidths. While this is large, it is conceivable that some there is some 'quantum interference', in which the total absorption probability arises from the coherent sum of absorption amplitudes, leading to interference between the individual atomic resonances lines. [12–16].

To begin, we briefly review how quantum interference between transitions to two excited states can modify the absorption spectrum [14, 15, 17, 18]. We do this because though, at face value, a realistic atomic model appears to meet the conditions for exhibiting quantum interference, it turns out that orthogonality of the Clebsch-Gordan and Wigner 6-j coefficients causes interference terms to vanish. Physically, this is a consequence of the fact that the emission mode-function from the hyperfine manifolds are orthogonal, so that the decay channels are in fact distinguishable.

Consider a toy model of an atom, consisting of a ground state $|1\rangle$, two excited states, $|3\rangle$ and $|4\rangle$, and potentially another low energy state, $|2\rangle$ into which the excited states may decay, as shown in Supplementary Figure 10. The atom is driven by a laser of frequency ω, which is detuned from level $|i\rangle$ by an amount $\Delta_i = \omega_i - \omega$. Note that $\Delta_4 - \Delta_3 = \omega_4 - \omega_3 \equiv \delta$.

The absorption profile, measured using the driving laser exhibits two pronounced resonances, when $\omega = \omega_i$, i.e. when $\Delta_3 = 0$ and when $\Delta_4 = 0$. Away from resonance, the absorption profile depends on whether the atomic decay from the excited state manifold is distinguishable or not. The critical distinction is whether the dipole operators add coherently or incoherently. We capture both scenarios in a single model, where $\alpha = 1$ for distinguishable decay and $\alpha = 0$ for indistinguishable decay, by considering a master equation for the density matrix of the atomic system

$$\dot\rho = -i[H,\rho] + \alpha\sum_{g,e}\mathcal{D}[\sqrt{\Gamma}\sigma_{ge}]\rho + (1-\alpha)\mathcal{D}[\sum_{g,e}\sqrt{\Gamma}\sigma_{ge}]\rho, \tag{13}$$

where $g \in \{|1\rangle, |2\rangle\}$, $e \in \{|3\rangle, |4\rangle\}$, H is the atomic Hamiltonian, $\sigma_{ij} = |i\rangle\langle j|$ and $\mathcal{D}[x]\rho = x\rho x^\dagger - (x^\dagger x\rho + \rho x^\dagger x)/2$ is the Lindblad superoperator. In a suitable rotating frame, the Hamiltonian is

$$H = \Delta_3|3\rangle\langle 3| + \Delta_4|4\rangle\langle 4| + (\Omega_3\sigma_{13} + \mathsf{H.c.})/2 + (\Omega_4\sigma_{14} + \mathsf{H.c.})/2,$$

where $\Omega_j = E_{\rm las}d_{1j}$ are the Rabi frequencies, depending on the laser amplitude and the dipole moment between $|1\rangle$ and $|j\rangle$.

The dynamical equations for the coherences contain the ingredients for possible interference. This can be seen for instance in the expression for ρ_{13}:

$$\begin{aligned}\dot\rho_{13} = (i\Delta_3 - \Gamma/2)\rho_{13} &- (1-\alpha)\Gamma\rho_{14}/2\\ &+ i\Omega_3(\rho_{11}-\rho_{33})/2 - i\Omega_4\rho_{43}/2.\end{aligned} \tag{14}$$

As we discuss below, it is the second term in Supplementary Equation 14 that gives rise to quantum interference effects in the absorption spectrum. If $\alpha = 0$, then the decay channels induce a coupling between the coherences ρ_{13} and ρ_{14} (and vice versa).

To see this, we first adiabatically eliminate coherences, which we implement by setting time derivatives of off-diagonal elements in ρ to zero, i.e. $\dot\rho_{ij} = 0$. We then solve the resulting algebraic equations for the coherences ρ_{ij} in terms of the populations ρ_{kk}. The absorption rate of photons from the probe laser is given by

$$A = i\Omega_3(\rho_{31}-\rho_{13})/2 + i\Omega_4(\rho_{41}-\rho_{14})/2. \tag{15}$$

Keeping the lowest order terms in Ω_j, and expanding the residues of the poles for small Γ gives a simple and accurate approximation to the absorption profile

$$A = \frac{\Gamma\Omega_3^2}{\Gamma^2+4\Delta_3^2} + \frac{\Gamma\Omega_4^2}{\Gamma^2+4\Delta_4^2} - \frac{2(1-\alpha)\Gamma(\Gamma^2-4\Delta_3\Delta_4)\Omega_3\Omega_4}{(\Gamma^2+4\Delta_3^2)(\Gamma^2+4\Delta_4^2)}. \tag{16}$$

Importantly, if the decay channels are distinguishable ($\alpha = 1$), then the absorption rate is just the sum of two lorentzian terms centred on each of the two transition frequencies, and is never zero. Conversely, if the channels are indistinguishable ($\alpha = 0$) there is an additional contribution of opposite sign, which, amongst other effects, causes the absorption to vanish in between the resonances. Clearly the interference term in Supplementary Equation 16 arises from the second term in Supplementary Equation 14.

We now turn to the more realistic model of a Cs atom with degenerate hyperfine manifolds, illustrated in Supplementary Figure 11. The master equation is given by

$$\dot{\rho} = -i[H,\rho] + \Gamma\left(\frac{2J_e+1}{2J_g+1}\right)\sum_q \mathcal{D}[\Sigma_q]\rho, \quad (17)$$

H describes the atomic energy levels and laser driving, $J_{e,g}$ are the total electronic angular momentum quantum number for the ground and excited state manifolds (for the Cs D_1 transition, $J_e = J_g = 1/2$),

$$\Sigma_q = \sum_{F_g,m_g,F_e,m_e} g_{F_g,m_g,F_e,m_e,q}|F_g m_g\rangle\langle F_e m_e|, \quad (18)$$

depends on the orbital quantum number F_j (which define the hyperfine manifolds) and the projection m_j (which label the substates within each manifold), and

$$g_{F_g,m_g,F_e,m_e,q} = (-1)^{F_e+J_g+1+I}\sqrt{(2F_e+1)(2J_g+1)} \times\langle F_g m_q|F_e m_e;1q\rangle \left\{\begin{array}{ccc} J_e & J_g & 1 \\ F_g & F_e & I\end{array}\right\} \quad (19)$$

depends on the Clebsch-Gordan coefficients $\langle F_g, m_q|F_e, m_e; 1q\rangle$, and the Wigner 6-$j$ symbol $\{...\}$ [18], as well as the nuclear quantum number I. Note that $q \in \{-1,0,1\}$ labels the three components of the spherical basis vectors [18].

As for the toy model above, the presence or absence of quantum interference depends on whether the master equation couples coherences $\rho_{F_g m_g,F_e m_e}$ and $\rho_{F_g m_g,F'_e m'_e}$ (with $F_e \neq F'_e$). Orthogonality relations satisfied by the Clebsch-Gordan coefficients, and by the $6-j$ symbols, yields the identity:

$$\sum_q \Sigma_q^\dagger\Sigma_q = \left(\frac{2J_e+1}{2J_g+1}\right)\sum_{F_e,m_e}|F_e m_e\rangle\langle F_e m_e|. \quad (20)$$

Writing out the components of the master equation for coherence between an arbitrary ground state $|F_g m_g\rangle$ and an arbitrary excited state $|F_e m_e\rangle$ gives:

$$\dot{\rho}_{F_g m_g,F_e m_e} = (i\Delta - \Gamma/2)\rho_{F_g m_g,F_e m_e} + \begin{array}{c}\text{driving}\\ \text{terms}\end{array}, \quad (21)$$

in exact analogy with Supplementary Equation 14 (we have suppressed the details of the driving terms, see [Eq. 7.530] of [18] for full details). Crucially, the RHS of Supplementary Equation 21 does *not* depend on $\rho_{F_g m_g,F'_e m'_e}$ for $F'_e \neq F_e$. That is, the decay channels do *not* induce any coherence between the different excited hyperfine manifolds. This result is ultimately a consequence of Supplementary Equation 20, the RHS of which is (proportional to) a projector onto the excited states, which, being diagonal, cannot introduce couplings between the excited states.

This formal argument demonstrates that in the case of the absorption spectrum of a single probe beam, there are no quantum interference terms to consider: effectively, we take $\alpha = 1$ in Supplementary Equation 16.

Physically, this corresponds to the fact that absorption of light from the probe beam necessarily drives an atom from one of its ground states $|g_i\rangle$ into one or other of the excited states, $|e_j\rangle$. Since the excited states are orthogonal, they are distinguishable (this is true even within an excited state manifold). When the atom ultimately relaxes back to a ground state $|g_k\rangle$, it emits light into some electromagnetic mode-function, $\psi_{kj}(\mathbf{r})$ depending on j and k. Since two different excited states $|e_j\rangle$ and $|e_{j'\neq j}\rangle$ are orthogonal, it follows that the mode-functions $\psi_{kj}(\mathbf{r})$ and $\psi_{kj'}(\mathbf{r})$ must also be orthogonal. Thus, after the atom has absorbed light from the probe, and then emitted light during relaxation, the final state of the atom-plus-field depends on which intermediate excited state the atom was in. As result, there can be no interference between the absorption spectra of each hyperfine manifold.

We note that the experiments in which quantum interference is seen typically employ multiple beams in a Raman configuration [12]. This allows atoms to coherently pass through several intermediate states and return to their original ground state, and so the final state of the atom-plus-fields have no record of which 'path' through the excited state manifold the atom took.

SUPPLEMENTARY METHODS

Apparatus

Light Source: A dual-beam linear absorption spectrometer operating at 895 nm was built using a pair of extended cavity diode lasers (ECDLs). The first of these, the master laser, is locked to the $6S_{1/2}$ $F = 4$ to $6P_{1/2}$ $F = 3$ transition of the $D1$ line in Cs at 894.6054 nm. This provided an optical reference frequency with a square-root-Allan-variance (SRAV) of ∼2 kHz for timescales between 1 s and 30 s, as shown by the grey circles on Supplementary Figure 1. These are the relevant time-scales for the spectroscopy, reflecting the time to take a single scan. The other laser is frequency locked to the master at a user-selectable frequency offset by stabilising the heterodyne beat frequency against a tunable radio frequency (RF) reference oscillator. This frequency lock contributed no additional frequency instability to this slave laser (red triangles). The resulting slave laser frequency stability is shown by the olive trace on Supplementary Figure 1. The RF reference oscillator was step tuned under computer control to ensure that the transmission data was synchronous with the selected frequency. The wavelength of the slave laser was centered on the twin absorption peaks at 894.5793 nm, corresponding to the $6S_{1/2}$ $F = 3$ to $6P_{1/2}$ $F = 3$ and $F = 4$ transitions. The offset frequency

was tuned in increments of 50 MHz over a 6500 MHz span that captured the atomic absorption features.

The slave laser output was coupled into a Fabry-Perot resonator whose length was actively controlled to keep it resonant with the slave laser light. The output of the cavity was coupled into an acousto-optic modulator (AOM) which was used to actively stabilise the power in the deflected beam. The output of the AOM was coupled into a single-mode fibre for spatial filtering and for delivery into the vacuum chamber. This fibre contained an auxiliary detector on a fibre coupler which provided the signal to stabilise the input power into the chamber. The rest of the light in the fibre was sent to a Cs sample cell embedded in a thermal and magnetic shield.

Spontaneous Emission: In a separate experiment the slave laser light was tuned to the centre of the atomic resonance and passed through a heated Cs cell for which the optical depth was 30 i.e. $\mathcal{T}_{\rm at} \approx e^{-30} \approx 10^{-12}$. A measurement of the residual transmission demonstrated that the laser output contained 1.6% broadband emission at its operating point.

The laser light was filtered using the fundamental mode of a scannable Fabry-Perot cavity ($\mathcal{F}$ =305 and free-spectral range 17.6 GHz). This was necessary because broadband emissions contaminate the measurement by creating a transmission offset associated with the non-resonant photons. Low level feedthrough of light from higher-order spatial modes of the optical cavity ($\sim$ 20% of the main mode) was heavily attenuated by coupling the beam into a single-mode optical fibre prior to introduction into the evacuated chamber.

The combined cavity and single-mode filtering provides a reduction in the spontaneous emission by a factor $2\mathcal{F}/\pi \approx 195$. We numerically modeled the effect of the residual spontaneous emission (0.008%) on the measured Cs spectrum and found that the perturbation to the fitted atomic Doppler width ν_D was only 1.8 ppm (equivalent to 3.6 ppm in $u_r(k_{\rm B})$). This systematic error can easily be reduced to below the 1 ppm level by a cavity finesse of over 1000.

Instrumental linewidths: The probe laser spectral lineshape can be expressed to a high degree of accuracy as a Voigt function [1] with a Lorentzian half-width-at-half maximum of $\Gamma_L^{\rm las}$ and Gaussian e^{-1}-halfwidth of $\Gamma_D^{\rm las}$. When this instrumental function is convolved with the atomic absorption profile (which, to first order, is also a Voigt function with parameters Γ_L and Γ_D), the apparently observed atomic lineshape is also a Voigt function with Lorentzian and Gaussian components given by $\Gamma_L = \Gamma_L^{\rm at}+\Gamma_L^{\rm las}$ and $\Gamma_D = \sqrt{(\Gamma_D^{\rm at})^2 + (\Gamma_D^{\rm las})^2}$, respectively.

The power spectral density (PSD), $S_\nu(f)$, of the probe laser frequency noise was directly measured by using the frequency-dependent absorption on the side of an atomic transition. Two regions of the PSD can then be identified [1]; at higher Fourier frequencies we see a dominant white frequency process, while at lower frequencies we observe steeper noise which in our case has a dominantly flicker ($1/f$) character. White noise components (with amplitude S_0) produce a Lorentzian lineshape with HWHM given by $\pi S_0/2$. The integrated probe laser noise at lower frequencies give rise to a Gaussian lineshape which has an estimated e-fold width of $\Gamma_D^{\rm las} = 0.88(39)$ MHz when integrated over the 40 ms observation time for each frequency point in the scan.

For correlated intensity and frequency noise the laser lineshape would be asymmetric and no longer be well-described by a Voigt profile. We measured the amplitude noise and found that the relative intensity noise was at least 30 dB lower than the phase noise of the laser from DC to 1 MHz. This implies that the frequency noise is dominant in determining the laser lineshape and that the lineshape is negligibly asymmetric. We also briefly note that a slight asymmetry would lead to a uniform shift of the measured atomic lines relative to their true optical frequency. This has no impact, to lowest order, on the retrieval of the Doppler width or the hyperfine separation frequency.

Optical power control: The spectrally purified light is passed through an acousto-optic modulator (AOM) and the deflected beam is coupled into a non-polarising fiber splitter, which splits the power in the ratio 9:1. The lower power beam is measured on a photodiode and a feedback loop adjusts the AOM input to maintain a constant optical power in the fibre. The higher power output is out-coupled and used for spectroscopy. The polarisation of this beam is fixed by passing it through a Glan-Taylor prism, and then a Wollaston beamsplitter is used to divide it into a reference and sample beam. The sample beam passes through the Cs vapour and common-mode amplitude variations in this signal are removed by dividing it by the reference beam signal.

Conventional thermometry: The Brewster-angled Cs sample cell is embedded in a cylinder of copper inside a multilayered thermostat, which is itself inside an evacuated chamber. The temperature of the thermostat can be tuned using a thermo-electric cooler attached to the outer-most layer of shielding. The temperature at the cell was monitored using a capsule-type standard platinum resistance thermometer (CSPRT) that was calibrated to the ITS-90 temperature scale with a temperature precision of better than 0.5 ppm in the range between 273 K and 300 K. However, our resistance meter (HP Model 3458A) limited our temperature measurement uncertainty to 1.2 ppm. Measurements using a second calibrated CSPRT located in the copper cylinder at a distance $\sim$ 50 mm away from the primary CSPRT revealed no temperature gradients above the 1.2 ppm level.

The ITS-90 temperature scale, T_{90}, itself is known to deviate from the true thermodynamic temperature, T by $T - T_{90} = 3.2\pm0.4$ mK at $T_{90} = 296$ K[2]. When the uncertainty in this correction is included, the total un-

certainty due to conventional thermometry is 1.9 ppm.

Measurement of residual B fields: Of the allowed Zeeman transitions in the D1 manifold of lines, the worst case susceptibility to an external magnetic field of 1.41 MHz/G was realised by the π transitions for $|m| = 3$. We designed a dual-layered coaxial shield manufactured from high-permeability alloy to attenuate the measured ambient field strength at the location of the experiment of less than 0.5 G by a factor of at least 31600 over the volume occupied by the Cs cells. The design was numerically verified using a magneto-static model implemented in a commercial finite-elements analysis package. Separately, we experimentally determined a minimum shielding factor of 1600 by applying a slowly modulated current at 7 Hz to a solenoid surrounding the magnetic shield. This measurement was limited by the sensitivity of the magnetic field sensor (0.01 G) – the maximum applied field needed to stay below the saturation field strength ($\sim$ 10 G) of the outer shield layer. The measured minimum shielding factor guaranteed that the worst-case Zeeman splitting remained below $1/10^{\text{th}}$ of the Lorentzian linewidth.

Optical detection: Reverse-biased silicon photodiodes were used to measure the optical power in each of the reference and sample beams. The photosignals were measured by digital multimeters (DMMs) recording the voltage generated by the photocurrent passing through a load resistor. The noise contributions to each measurement of the atomic transmission from shot noise, Johnson noise and the DMMs are shown in Supplementary Figure 2 as a function of input signal level to each photodiode (the stair-case structure revealed in the figure arises from a change in the DMM range). It is clear that the transmission measurement are shot-noise limited in several bands of powers that lie just near the maximum of each range. At the highest powers used in the experiments ($\sim$0.6μW), shot noise contributed more than 50% of the total measurement noise.

The linearity of the photodetection chain was measured in two ways. In the first approach, the ratio of the transmitted and incident detector signals were measured over the full range of common-mode input power variation. This provided a reliable calibration curve for which the deviations from linearity at high powers could then be corrected. For low powers, this method did not provide sufficient signal-to-noise for calibration. Instead, we monitored the ratio of absorption coefficients for the two optical transitions. The second approach relies on the fact that the Clebsch-Gordon coefficients for these transitions require that the linear (i.e. low-power) ratio of absorption coefficients should be 1:3 [3]. Forming this ratio, and enforcing that the extrapolation to zero power agrees with the 1:3 condition, we have a guaranteed, *in situ* and independent way to determine any nonlinearity in the detector response at low powers. In this way, we were able to resolve and remove the nonlinearities in the photodetection scheme. The technique allowed us to measure offsets corresponding to <8 pW allowing us to ensure linearity over a dynamic range of $10^5 : 1$. A more complete description of this method is found in Ref. [4].

Etalon reduction: Here we estimate the levels of stray reflections, specular and diffuse, required to cause the largest of the etalons observed in the data (Fig. 1 of main article), which have an amplitude of 30 ppm in the transmission ratio $\mathcal{T}$ over the $\sim$ 6 GHz spectral band we sampled. These etalons appear in spite of some substantial efforts taken in the experiment to eliminate them; we will demonstrate that very modest amounts of stray light will lead to these levels of baseline imperfections and are difficult to completely eliminate under these high signal-to-noise detection scenarios.

We model etalons formed by specular reflections as Fabry-Perot cavities in the low finesse limit. In this case, the transmission is given by [5]:

$$\mathcal{T}_e = \frac{\mathcal{T}}{1 + (2\mathcal{F}/\pi)^2 \sin^2(\pi f/f_{\text{FSR}} + \phi)} \tag{22}$$

$$\approx \mathcal{T}\left(1 - a\sin^2(\pi f/f_{\text{FSR}} + \phi)\right) \quad \text{for } \mathcal{F} \ll 1 \tag{23}$$

where $a = 4\mathcal{F}^2/\pi^2$ is the etalon amplitude, and $\mathcal{T}$ is the peak transmission, f_{FSR} is the free spectral range, ϕ is a phase offset and $\mathcal{F} = \pi\sqrt{R}/(1-R)$ is the finesse of a resonator composed of optical elements each with power reflectivities of value R. Two parallel specular surfaces, each of reflectivity 0.003%, is sufficient to cause the etalon with a =30 ppm.

To minimise specular reflections, we used anti-reflection coated polarisation optics (Thorlabs Glan-Taylor GT10-B polarising prisms and Wollaston WP10-B polarising beamsplitters) with specified reflectivities of less than 0.2% at 895 nm. These optics were also intentionally misaligned from normal incidence as much possible whilst still satisfying their input angle tolerance to further reduce etaloning. Reflective optics were used after the polarising beamsplitters to steer and focus the beams onto the photodetectors avoiding elatons in refractive optics.

In an initial assessment of optical alignment, we swept the probe laser through a range of more than 50 GHz far away from the Cs resonances (Supplementary Figure 3). This sweep was performed by slowly tuning the probe laser whilst the offset lock was disabled, and its optical frequency was monitored using a commercial wavemeter (High Finesse WS/7) with a precision of 10 MHz.

In this diagnostic configuration, two etalons with periods 3.14(1) GHz and 37.86(1) GHz with amplitudes 400 ppm and 800 ppm, respectively, were visible (Supplementary Figure 3). The 3.1 GHz etalon was easily determined to have come from a mal-aligned Glan-Taylor polarising prism and this was remedied by realignment of that optic. This etalon corresponded to a resonator finesse of $\mathcal{F} \approx 0.031$ and reflectivity of $R = 0.01\,\%$ off

each reflective surface. We note that such prisms often have a limited beam acceptance angle, typically $\sim 5^\circ$ away from normal incidence, that makes it difficult to severely deflect the retro-reflected beam away from its input path. We were therefore forced to rely on the performance of the anti-reflection coating. In the final configuration, we opted for a compromise between the efficacy of the polariser and the generation of unwanted etalons by slightly angling the optic away from normal incidence. The etalon was replaced with a slowly varying background that is likely due to a residual polarisation modulation introduced by the intentional misalignment into the polarising prism prior to the polarising beam-splitter.

A broader etalon with period ~38 GHz was also evident. This was found to be caused by the Cs cell windows and was experimentally confirmed by temporarily directing the s-polarised output of the beamsplitter to the cell to increase the expected reflectivity from near zero for the p-polarisation to 16.4% for the $s-$polarisation input at the Brewster input angle $\theta = 56.9^\circ$. This produced an exaggerated modulation with amplitude ~200 000 ppm as shown in Supplementary Figure 3 (amber trace).

We now consider the effect of slight alignment and polarisation perturbations away from the Brewster condition and the typical size of the etalon this could cause. To completely extinguish the reflection off a Brewster-angled surface when the input beam is purely linearly polarised it must not only be incident at the Brewster angle, it needs to be purely in the p-polarisation as defined by the plane of the cell window. Suppose that the input polarisation axis forms an angle ϕ with respect to the p-plane defined by the cell window.The total reflectivity R is the sum of the s- and p-component reflectivities, *i.e.*

$$R = (r_s \sin\phi)^2 + (r_p \cos\phi)^2 \tag{24}$$

$$= r_s^2 \sin^2\phi \tag{25}$$

where $r_s = \sqrt{0.164}$ and $r_p = 0$ are the amplitude reflectivities at the Brewster condition $\theta = 56.9^\circ$. The etalon amplitude a can be parameterised as a function of the angle ϕ by substitution of Supplementary Equation 25 into Supplementary Equation 23:

$$a = \frac{4\mathcal{F}^2}{\pi^2} \tag{26}$$

$$= \frac{4r_s^2 \sin^2\phi}{(1 - r_s^2 \sin^2\phi)^2} \tag{27}$$

$$\approx 4r_s^2\phi^2 \qquad \text{for } \phi \ll 1. \tag{28}$$

An ability to individually rotate each cell around its optical axis, or to precisely rotate the beam polarisation without introducing extra etalons, would be convenient methods to ensure that the polarisation alignment criterion is met. Unfortunately, this capability was missing from the design of the thermal isolator.

Applying the Fresnel relations [6], we can determine the maximum etalon amplitude caused by a deviation of the input beam from the Brewster angle. Since the 3.25 mm diameter beam needed to pass through the 20mm diameter apertures separated by 300mm on the outer thermal shield, this constrained the maximum angular deviation of the beam from Brewster's angle to be $\sim 3^\circ$, causing a $R = 0.1\%$ reflection and 4400 ppm amplitude etalon. We note that even a $\phi = 0.4^\circ$ misalignment is sufficient to cause an etalon with 30 ppm amplitude.

The transmission background was remeasured after optimising the optics (red trace in Supplementary Figure 3), showing that the two etalons previously identified have been suppressed and the maximum variations were approximately 1000 ppm over 30 GHz. The lack of strong periodicity suggested that the residual etalons arising from the polarisation and input beam misalignments were small compared to some slow, frequency-dependent variation of the optical split ratio introduced by perhaps the tilted GT prisms. During the actual experiment, the scan range was only 6 to 12 GHz away from the reference laser (denoted by 0 GHz detuning on Supplementary Figure 3). We expect the typical variations to be reduced to $\sim$ 170 ppm over this smaller frequency interval. Residual etalons at the 30 ppm level were still evident.

We now consider non-specular sources of etalons. An etalon may be formed by scattered light that subsequently re-enters the beam path and becomes co-incident on a photodiode. We model this type of interference by superposing the scattered field E_s that travels an additional path length δL with respect to a gaussian beam path by forming the overlap integral

$$\mathcal{T}(\lambda) = \int_{-\infty}^{\infty} dr\, |\mathcal{I}(r)|^2 \tag{29}$$

$$\mathcal{I}(r) = \int_{-\infty}^{\infty} dr' \left(\sqrt{\xi}\tilde{E}_s(r';\lambda) + \tilde{E}_g(r';\lambda)\right) \tag{30}$$

where ξ is the ratio of the collected scattered optical power to the beam power, E_g is the electric field of the gaussian beam. The electric fields are given by:

$$\tilde{E}_s(r';\lambda) = E(r';w_s)e^{-i\frac{2\pi(r-r')^2}{2\lambda(L+\delta L)}}e^{-i2\pi\frac{\delta L}{\lambda}} \tag{31}$$

$$\tilde{E}_g(r';\lambda) = E(r';w_g)e^{-i\frac{2\pi(r-r')^2}{2\lambda L}} \tag{32}$$

where $E(r;w) = (w\sqrt{\pi})^{-1}\exp(-(r/w)^2)$ is the transverse amplitude profile with width w, L is path length taken by the gaussian beam and δL is the path length difference traveled by the scattered light. We used typical values $w_s = 20\,\mu\text{m}$, $w_g = 2\,\text{mm}$, $L = 60\,\text{cm}$ and $\delta L = 20\,\text{cm}$.

An interference with 0.1 ppb of the optical power (*i.e.* 10 ppm in electric field amplitude) in the beam is sufficient to produce etalons with amplitude $a = 30\,\text{ppm}$. This level of collected scatter can be from many potential

sources; the total scattered fraction $\mathcal{S}$ from the complement of the specular reflectance at normal incidence is related to the rms surface roughness σ by[7]:

$$\mathcal{S} = R_0(1 - e^{-(4\pi\sigma/\lambda)^2}) \tag{33}$$

$$\approx R_0\,(4\pi\sigma/\lambda)^2 \tag{34}$$

where R_0 is the reflectance of a perfectly smooth surface composed of the same material and $\lambda \gg \sigma$ is the optical wavelength. Super-polished laser mirrors ($R_0 \sim 1$) have typical roughness $\sigma \sim 0.1$ nm which leads to $\sim$2 ppm of scattering loss at 895 nm. Even for a modest roughness of $\sigma = 3$ nm the scatter becomes 2000 ppm, which is easily resolvable at our signal-to-noise levels. An etalon with $a = 30$ ppm will occur if the surface of the reference-arm photodiode back-scatters approximately 200 ppm of the input beam, and that part of this scatter is collected by the probe-arm photodiode by a specular reflection off the Wollaston beamsplitter. For the largest amplitude etalon, the inferred etalon length ($\sim$12 cm) between the scattered light and the probe beam is consistent with the fringe spacing ($\sim$ 1.5 GHz) seen in Fig. 1 of the main article. It is difficult to further mitigate etalons in the apparatus given the very low levels of scatter required to produce measurable background undulations.

Etalon fitting: After etalon contributions have been reduced by physical means, it remains to account for residual etalon effects by including suitable terms in the model used to fit the data. To this end, we include etalon terms, as described in the main text (see Equation 1 therein). Here, we describe the fitting methodology, and analyse its stability against comparable data sets and its robustness against overfitting.

We begin the fitting process by fitting a model that ignores etalons, $N_{\rm et} = 0$, for which the residuals are shown in the left-hand column of Supplementary Figure 5, and exhibit strong oscillatory features far from the resonances (indicated by the blue shaded regions). Since we don't know *a priori* how many etalons may be contributing to these features, we perform independent fits with different numbers of etalons, $N_{\rm et}$, allowing $N_{\rm et}$ to vary from 1 to 5. For each $N_{\rm et}$, we allow all etalon parameters, f_j, a_j, and ϕ_j to be free fitting parameters, where $j \in \{1, ..., N_{\rm et}\}$ labels each etalon. When $N_{\rm et}$ is large enough to have accommodated all significant etalons, the residuals should be consistent with noise. For each value of $N_{\rm et}$, we extract a complete set of fitting parameters, including the fitted doppler width, $\Gamma_D^{\rm fit}$.

To demonstrate the stability of this approach when fitting to comparable sets of data, we have taken the 200 scans at highest power, partitioned them into four blocks of 50 scans, and averaged each block of scans. We apply the fitting procedure described above to each of these four block-averaged scans, and extract a set of etalon parameters for $N_{\rm et} = 1, ..., 5$. Within each block Supplementary Figure 4 shows the fitted etalon FSR f_j (top row), and amplitude a_j (bottom row), for $j \in \{1, ..., N_{\rm et}\}$.

If the underlying etalons responsible for the structure in the residuals were stable over the 200 scans, then we would expect to find agreement between fitted etalon parameters when comparing different blocks. This is borne out in Supplementary Figure 4: we see that the fitted FSRs (plotted as effective etalon lengths, $L = c/(2f_{\rm FSR})$) are largely consistent across blocks, and these are stable as $N_{\rm et}$ increases, e.g. all blocks are well fitted by the largest etalon with an effective length of ~ 13 cm and amplitude $\sim 30 - 35$ ppm (blue points), and another of effective length ~ 4 cm and amplitude $\sim 25 - 30$ ppm (orange points). For small $N_{\rm et}$, Block 1 exhibits some $N_{\rm et}$-dependence in the fitted FSR and amplitude of the $j = 2$ etalon (orange points), whilst the fitted amplitude of the $j = 4$ etalon is notably higher in Block 1 than in the other blocks (red points). Otherwise, the fitted parameters are in good agreement between blocks. We also note that the fitted phases, ϕ_j (not shown), are likewise consistent between blocks.

The residuals between the block-average data and the fitted functional form are shown in Supplementary Figure 5 for $N_{\rm et} = 0, ..., 5$ etalons. With zero etalons included in the model, strong oscillatory features are notable (first column of Supplementary Figure 5). These are reduced after including a single etalon (second column of Supplementary Figure 5), and improve as $N_{\rm et}$ increases. After $N_{\rm et} = 2$ or 3 etalons are accounted for, the residuals are close to the underlying noise (though with a persistent, unexplained tick at the high frequency end).

Finally, for each block, and for each $N_{\rm et}$, we extract the fitted value for $\Gamma_D^{\rm fit}$. Supplementary Figure 6 shows the difference between $\Gamma_D^{\rm fit}$ and $\Gamma_D^{\rm fit}$ measured independently with calibrated PRTs, for successively larger $N_{\rm et}$. Each block is distinguished by different colours and symbols (see caption), and the mean over the blocks is shown as hollow circles. Clearly, the systematic difference between $\Gamma_D^{\rm fit}$ and $\Gamma_D^{\rm fit}$ for each $N_{\rm et}$ behaves consistently across all blocks, starting at around 100 ppm for $N_{\rm et} = 0$ (i.e. no etalons), and decreasing thereafter. Most importantly, at $N_{\rm et} = 2$, $\Gamma_D^{\rm fit}$ becomes statistically indistinguishable from $\Gamma_D^{\rm PRT}$, and remains so as $N_{\rm et}$ increases further.

In summary, we see from Supplementary Figure 5 that three etalons are sufficient to account for almost all of the systematic features observed in the fit residuals, and we see from Supplementary Figure 6 that no more than two etalons are needed to obtain good agreement between $\Gamma_D^{\rm fit}$ and the independently measured $\Gamma_D^{\rm PRT}$. This analysis engenders confidence that the inclusion of relatively few of the most significant unwanted etalons in the fitting model is sufficient to account for their systematic influence on $\Gamma_D^{\rm fit}$ and explain the features seen in the fit residuals.

For reference, Supplementary Figure 7 shows the absolute values $\Gamma_D^{\rm PRT}$ for each scan. They were all taken at nominally 296 K, with the greatest rate of change being 2.2 ppm/min.

SUPPLEMENTARY REFERENCES

[1] G. D. Domenico, S. Schilt, and P. Thomann, Appl. Opt. **49**, 4801 (2010).
[2] J. Fischer, M. Podesta, K. Hill, M. Moldover, L. Pitre, R. Rusby, P. Steur, O. Tamura, R. White, and L. Wolber, International Journal of Thermophysics **32**, 12 (2011).
[3] D. Steck, "Cesium D line data (unpublished) `http://steck.us/alkalidata`," (2009).
[4] Manuscript in preparation (2014).
[5] A. Siegman, *Lasers* (University Science Books, 1986).
[6] B. Saleh and M. Teich, *Fundamentals of Photonics*, Wiley Series in Pure and Applied Optics (Wiley, 2013).
[7] H. E. Bennett and J. O. Porteus, J. Opt. Soc. Am. **51**, 123 (1961).
[8] L. Young, W. T. Hill, S. J. Sibener, S. D. Price, C. E. Tanner, C. E. Wieman, and S. R. Leone, Phys. Rev. A **50**, 2174 (1994).
[9] J. M. Amini and H. Gould, Phys. Rev. Lett. **91**, 153001 (2003).
[10] C. Vadla, V. Horvatic, and K. Niemax, Applied Physics B **84**, 523 (2006).
[11] T. M. Stace and A. N. Luiten, Phys. Rev. A **81**, 033848 (2010).
[12] M. Biercuk, H. Uys, A. VanDevender, N. Shiga, W. Itano, and J. Bollinger, Nature **458**, 996 (2009).
[13] R. C. Brown, S. Wu, J. V. Porto, C. J. Sansonetti, C. E. Simien, S. M. Brewer, J. N. Tan, and J. D. Gillaspy, Phys. Rev. A **87**, 032504 (2013).
[14] M. Horbatsch and E. A. Hessels, Phys. Rev. A **82**, 052519 (2010).
[15] M. Horbatsch and E. A. Hessels, Phys. Rev. A **84**, 032508 (2011).
[16] A. Marsman, M. Horbatsch, and E. A. Hessels, Phys. Rev. A **86**, 040501 (2012).
[17] D. A. Cardimona, M. G. Raymer, and C. R. S. Jr, Journal of Physics B: Atomic and Molecular Physics **15**, 55 (1982).
[18] D. A. Steck, http://steck. us/teaching (revision 0.8. 2, 6 December 2011) **46**, 55 (2007).
